# GPCRact: a hierarchical framework for predicting ligand-induced GPCR activity via allosteric communication modeling

**DOI:** 10.1093/bib/bbaf719

**Published:** 2026-01-15

**Authors:** Hyojin Son, Gwan-Su Yi

**Affiliations:** Department of Bio and Brain Engineering, Korea Advanced Institute of Science and Technology (KAIST), 291 Daehak-ro, Yuseong-gu, Daejeon 34141, Republic of Korea; Department of Bio and Brain Engineering, Korea Advanced Institute of Science and Technology (KAIST), 291 Daehak-ro, Yuseong-gu, Daejeon 34141, Republic of Korea

**Keywords:** G-protein-coupled receptors (GPCRs), allosteric communication, graph neural network, interpretable AI, drug discovery

## Abstract

Accurate prediction of ligand-induced activity for G-protein-coupled receptors (GPCRs) is a cornerstone of drug discovery, yet it is challenged by the need to model allosteric communication—the long-range signaling linking ligand binding to distal conformational changes. Prevailing sequence-based models often fail to capture these three-dimensional dynamics, a limitation frequently masked by averaged performance on simpler Class A targets. To address this, we introduce GPCRact, a novel framework that models the biophysical principles of allosteric modulation in GPCR activation. It first constructs a high-resolution, three-dimensional structure-aware graph from the heavy-atom coordinates of functionally critical residues at binding and allosteric sites. A dual attention architecture then captures the activation process: cross-attention encodes the initial ligand-protein interaction at the binding site, whereas self-attention learns the subsequent intra-protein signal propagation. This hierarchical architecture is built upon an E(n)-Equivariant Graph Neural Network (EGNN) to explicitly model conformational consequences of ligand binding, and is further refined with a tailored loss function and inference logic to mitigate error propagation. Underpinned by GPCRactDB, a comprehensive database we constructed for this study, GPCRact not only achieves state-of-the-art performance but also demonstrates robustly superior accuracy on a curated benchmark of allosterically complex receptors where existing models systematically underperform. Crucially, analysis of the learned attention weights confirms that the model identifies biologically validated allosteric pathways, offering a significant step toward resolving the black box nature of previous methods. Thus, GPCRact provides a more accurate, interpretable, and mechanistically-grounded solution to a long-standing challenge, paving the way for effective structure-guided drug discovery.

## Graphical Abstract

**Graphical Abstract:**
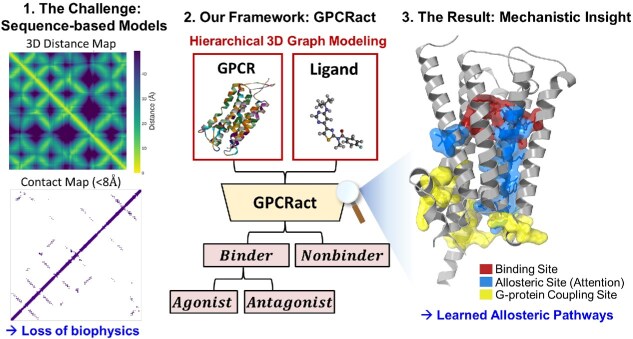

## Introduction

G-protein-coupled receptors (GPCRs) constitute the largest family of therapeutic targets, mediating a vast array of physiological processes and accounting for approximately 34% of all FDA-approved drugs [1]. However, their pharmacological complexity presents a significant bottleneck for traditional drug discovery, necessitating computational approaches to efficiently navigate the vast chemical space for lead identification and optimization [2]. Structure-based drug discovery has been transformed by recent breakthroughs in deep learning, notably AlphaFold2 [3] for protein structure prediction and AlphaFold3 [4] for modeling biomolecular interactions. Despite these advances, a critical challenge persists: predicting the functional consequences of ligand binding. GPCR function is governed not by static structures alone but by ligand-induced conformational dynamics and long-range allosteric pathways that link the binding pocket to distal intracellular signaling domains [5]. Consequently, accurately predicting ligand-induced GPCR activity—whether a compound acts as an agonist or antagonist—based on its receptor interaction remains a fundamental challenge in computational biology and drug design.

**Figure 1:**
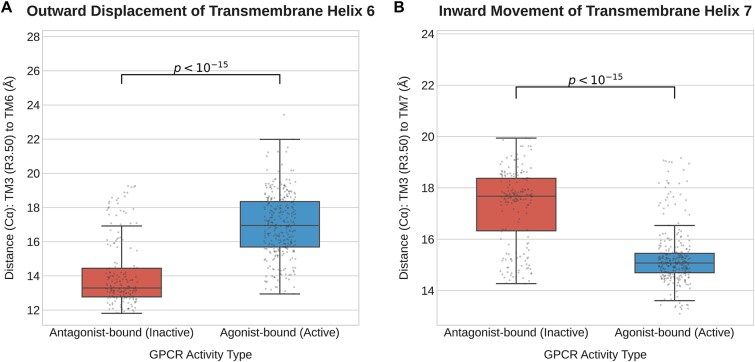
Conformational changes upon GPCR activation. Box plots comparing key inter-helical C$\mathrm{\alpha}$ distances between antagonist-bound (inactive) and agonist-bound (active) GPCR structures. (A) Outward displacement of transmembrane helix 6 (TM6) relative to TM3, measured from residue R3.50. (B) Inward movement of transmembrane helix 7 (TM7) relative to TM3, measured from residue R3.50. Statistical significance was determined by a two-sided Welch’s t-test.

Fundamentally addressing this challenge requires moving beyond a simplistic binary classification of active or inactive states to define the biophysical basis of GPCR activation. This functional state is not an abstract label but instead is defined by three-dimensional conformational changes at the Ångström-scale. Our analysis of hundreds of GPCR structures revealed a conserved biophysical signature of activation: significant outward displacement of transmembrane helix 6 (TM6) and corresponding inward movement of transmembrane helix 7 (TM7) (Fig. 1). Specifically, the C$\mathrm{\alpha}$ distance between key residues R3.50 and E6.30 increases from approximately 13 Å in the inactive state to 17 Å upon activation, creating the intracellular cavity for G-protein engagement. Conversely, the distance from R3.50 to Y7.53 decreases from approximately 18 Å to 15 Å, stabilizing the conserved NPxxY motif in its active conformation. These specific, measurable structural transitions constitute the physical hallmarks of allosteric activation [6]. This reframes the prediction task from a simple classification problem to one of modeling this precise conformational shift.

Existing computational paradigms for activity prediction, while advancing scalability and efficiency, are fundamentally disconnected from this biophysical principle. Predominant sequence-based models, such as DeepREAL [7] and AiGPro [8], are inherently limited by their reliance on one-dimensional representations, leading to three primary flaws. First, they discard the explicit 3D coordinates constituting the physical medium of allostery, resulting in a loss of the biophysical ground truth. Even sophisticated attempts to mitigate this, such as AiGPro’s use of multiple sequence alignments (MSAs) indexed by the Ballesteros-Weinstein (BW) system [9], cannot overcome this core limitation. Second, they create a representation mismatch—treating ligands at the atom level while abstracting the protein to the residue level—thereby precluding the model from learning physically meaningful interactions. Third, they operate as black boxes, offering no mechanistically interpretable insight into the allosteric pathway. In contrast, structure-based approaches, exemplified by the GPCR-IPL score [10], directly incorporate pocket-level features from 3D structures. However, their reliance on static snapshots renders them fundamentally unable to model the dynamics of activation—a critical failure to capture the conformational shifts essential to function. Furthermore, their dependence on a small set of resolved complexes leads to a lack of generalizability, severely restricting their utility for large-scale benchmarking. In essence, neither paradigm is designed to learn the process of allosteric activation. It is also crucial to distinguish our predictive framework from classical analytical frameworks for allostery. Biophysical methods, encompassing graph-theoretic approaches such as Protein Contact Networks (PCN) [11] and Protein Structure Networks (PSN) [12], as well as dynamic profiling techniques like Elastic Network Models (ENM) [13] and Perturbation Response Scanning (PRS) [14], are powerful analytical tools. They are designed to characterize the intrinsic, state-dependent dynamics and potential communication pathways within a single, known protein structure or conformational state. However, they are not predictive frameworks designed to classify the functional outcome (i.e. agonism or antagonism) resulting from an interaction with a novel, uncharacterized ligand.

To bridge this critical gap—the lack of a predictive framework that models ligand-induced allosteric communication—we introduce GPCRact, a mechanistically grounded framework that models the key biophysical principles governing GPCR activation: 3D structure, allosteric communication, and the conformational changes leading to a functional state. To address data fragmentation, we first constructed GPCRactDB, a comprehensive database integrating functional and structural information for hundreds of human GPCRs. Leveraging this database, GPCRact introduces three core innovations. First, it constructs a high-resolution structural graph using the heavy-atom coordinates of key residues to precisely capture atomic-level interactions. Second, a dual attention architecture emulates the biological hierarchy of activation: cross-attention models initial ligand-protein interactions at the binding site, while self-attention propagates the allosteric signal to distal domains. This architecture is complemented by a tailored loss function and an inference logic designed to mitigate error propagation across binding and activity predictions. Third, it employs an E(n)-equivariant graph neural network (EGNN) [15] that operates directly on atomic coordinates, preserving crucial geometric information to learn features representative of the structural changes inherent to activation. This integrated approach yields superior predictive performance, particularly for allosterically complex receptors where previous models have systematically underperformed. Moreover, analysis of learned attention weights confirms alignment with validated allosteric pathways. GPCRact thus offers a predictive framework that prioritizes both accuracy and mechanistic transparency, advancing structure-guided drug discovery for diverse GPCR targets.

## Materials and methods

### Construction of GPCRactDB

To build a unified resource for ligand-induced GPCR activity prediction, we constructed GPCRactDB by integrating functional annotations and structural data from public repositories, including DrugBank [16], GPCRdb [17], and ChEMBL [18]. This process involved developing a robust pipeline to annotate the mode of action (MoA) for each interaction. For unstructured bioassay descriptions, particularly those from PubChem [19], a dictionary-based named entity recognition (NER) process was employed to parse and classify activity data. To establish a clear functional cutoff for our classification task, all dose–response values (e.g. ${IC}_{50},{EC}_{50},{K}_i,{K}_d$) were standardized to $\mathrm{\mu} \mathrm{M}$, and a threshold of 10$\mathrm{\mu} \mathrm{M}$ was applied to distinguish binders (agonists or antagonists) from nonbinders. To ensure data consistency, all the compound and protein identifiers were mapped to standardized formats (InChIKey and UniProt accessions [20], respectively). Conflicting MoA annotations for a given ligand-GPCR pair were systematically resolved through a majority voting scheme, with the remaining conflicts addressed by manual curation (see Supplementary Methods; SM1).

Concurrently, structural entries were downloaded in mmCIF format from the RCSB PDB [21] and processed via Biopython [22]. For each entry, the primary GPCR chain was identified via local sequence alignment (Smith-Waterman [23] with the BLOSUM62 [24] matrix) against its corresponding UniProt sequence. Ligands were identified from HETATM records, filtered to remove nondrug-like molecules, and assigned standardized InChIKeys. The final step involved annotating each structure using our curated MoA data. Our classification focused on agonism and antagonism, as these two MoAs account for approximately 95% of approved GPCR-targeting drugs [17]. Details on the handling of all MoA categories (including partial agonists, inverse agonists, and allosteric modulators) are provided in SM 1.4. Additionally, structures determined in complex with auxiliary proteins were excluded to avoid induced-fit effects that would require separate modeling.

### Biophysical analysis of GPCR activation signatures

To define the structural hallmarks of GPCR activation, a systematic analysis was performed on the agonist-bound and antagonist-bound holo structures from GPCRactDB. For structural and sequential correspondence, all structures were superimposed on their transmembrane (TM) domains, and the residues were indexed using the BW numbering scheme retrieved from GPCRdb. The conformational change was quantified using two distinct distance metrics relative to the highly conserved R3.50 residue on TM3 [25]. The first metric, capturing the outward displacement of TM6, measured the C$\mathrm{\alpha}$ distance from R3.50 to the geometric centroid of key residues on TM6 (E/D6.30, R/K6.34, T/S6.48). The second metric similarly quantified the inward movement of TM7 by measuring the distance from R3.50 to the geometric centroid of key residues on TM7 (Y7.49, I/L7.50, Y7.53). The distributions of the agonist- versus antagonist-bound structures were then compared using a two-sided Welch’s t-test, as depicted in the Introduction (Fig. 1).

### The GPCRact modeling framework

#### Overall architecture

**Figure 2:**
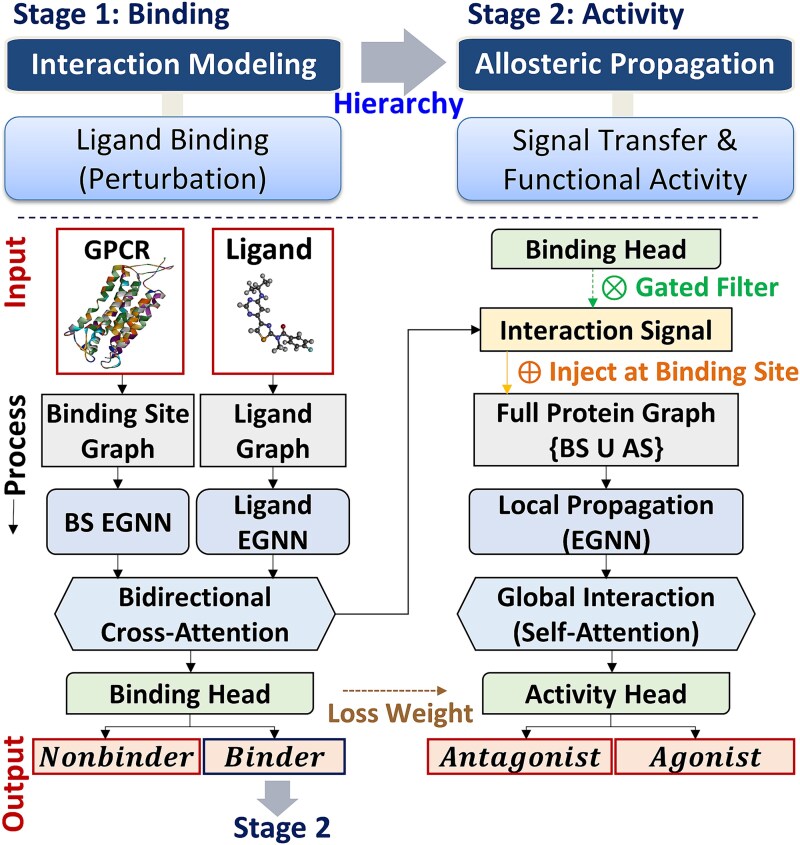
Hierarchical architecture of the GPCRact framework. The model predicts ligand-induced GPCR activity in a two-stage process. Stage 1 (binding): an interaction module uses E(n)-Equivariant graph neural networks (EGNNs) to encode ligand and binding site graphs. Bidirectional cross-attention then models their interaction to predict the binding status (binder/nonbinder) and generate an interaction signal. Stage 2 (activity): For the predicted binders, the interaction signal is injected into the full protein graph. An Allosteric propagation module, using local (EGNN) and global (self-attention) layers, then predicts the final functional activity (agonist/antagonist).

GPCRact is a two-stage, hierarchical deep learning framework designed to model the biophysical process of GPCR activation, from local ligand binding to global allosteric effects (Fig. 2). Its architecture comprises two specialized modules that operate sequentially. Stage 1, the Interaction Module, processes graphs of the ligand and the protein’s binding site to produce a binary prediction of the binding event and an interaction signal. The interaction signal consists of the binding site residues’ feature vectors updated via cross-attention with the ligand. In Stage 2, the Allosteric Propagation Module uses this signal to predict the ligand’s functional activity. The transfer of the interaction signal is controlled by a dynamic gating mechanism before being integrated with the full protein graph. The signal is subsequently propagated throughout the protein structure via a local EGNN and global self-attention [26] layers, enabling the model to learn the end-to-end allosteric communication pathway.

#### Input representation: atomistic structural graphs

GPCRact uses atomistic graph representations for both proteins $\left({G}_p\right)$ and ligands $\left({G}_l\right)$ to resolve the feature mismatch of prior models. To ensure computational tractability, the protein graph ${G}_p$ is constructed as a functionally critical subgraph. Specifically, we define two functional regions—a consensus Binding Site (cBS) and a consensus Allosteric Site (cAS)—and construct ${G}_p$ from their union. The cBS comprises all residues within 4.0 Å of a bound ligand, while the cAS consists of the top 100 residues ranked by their mean C$\mathrm{\alpha}$ displacement between the receptor's apo and holo states. These sites are derived from a comprehensive analysis of all available experimental holo structures for each receptor, establishing a stable and representative structural prior. We validated that this consensus-based definition is robust, as the sites are highly consistent across diverse ligand-bound structures (Fig. S1). Each node is described by a 37-dimensional feature vector encoding residue identity, site membership (cBS or cAS), atomic coordinates, and atomic role (C$\mathrm{\alpha}$ versus sidechain) (Table S2). For the final graph representation, nodes are instantiated at the C$\mathrm{\alpha}$ atom of each residue and supplemented with key functional sidechain atoms selected to capture specific chemical properties (Table S3). The edges in the graph connect the k-Nearest Neighbors (k = 64) for each node in 3D space (Table S4). The ligand graph$\left({G}_l\right)$ is a complete molecular graph constructed from a 3D conformer generated from its SMILES string via the ETKDG method [27]. Ligand nodes (heavy atoms) are characterized by a 19-dimensional vector encoding elemental and chemical properties, while edges represent covalent bonds.

#### Stage 1: interaction module for binding prediction

The Interaction Module computes a binary prediction for the binding event based on the initial protein-ligand recognition. It takes the atomistic graphs of the ligand$\left({G}_l\right)$ and only the protein’s binding site subgraph as input. First, separate EGNN encoders process each graph independently to generate initial 3D-aware node embeddings. A bidirectional cross-attention mechanism is then employed, where the protein binding site nodes and ligand nodes mutually update their representations by attending to each other’s features. The resulting context-aware embeddings for the binding site and the ligand are then pooled into fixed-size vectors, concatenated, and passed through a multilayer perceptron (MLP), termed the binding head, to yield a single logit for the binding prediction. The feature vectors of the protein binding site nodes, updated via this cross-attention process, constitute the interaction signal passed to Stage 2.

#### Stage 2: allosteric propagation module for activity prediction

The Allosteric Propagation Module is tasked with modeling the signal transduction process from the binding site to distal regions to predict the final functional outcome. The module first receives the interaction signal from Stage 1, which is dynamically modulated by the gating mechanism. Specifically, the signal magnitude is scaled by the binding probability predicted in Stage 1. This gated signal is then injected into the full protein graph$\left({G}_p\right)$ via an element-wise addition to the feature vectors of the binding site nodes. The propagation of this initial perturbation is modeled through a two-step process. First, a series of EGNN layers perform local, structure-aware message passing from the binding site to spatially adjacent residues. Following this, self-attention layers are applied to capture long-range, global interactions across the entire protein structure, yielding a final global conformational state representation. This representation is then pooled and passed to the activity head, an MLP that outputs the final logits for agonist versus antagonist classification.

### Model training and inference strategy

#### Hierarchical multitask training

GPCRact is trained using a hierarchical multitask strategy designed to optimize its two specialized modules concurrently. The framework jointly learns two distinct tasks: (i) a binary classification task for binding prediction, performed by the binding head, and (ii) a binary classification task for activity prediction (agonist versus antagonist), performed by the activity head. While the binding head is trained on all samples, the training of the activity head is conditional. Specifically, a loss mask is applied during each training step, ensuring that the activity loss$\left({\mathcal{L}}_{act}\right)$ is computed and backpropagated only for samples with a ground-truth label of binder. This approach specializes the activity head for its intended function—distinguishing between agonism and antagonism—without being confounded by nonbinding samples during training. The final objective is a composite loss, defined as the weighted sum of the losses from both tasks:${\mathcal{L}}_{total}={\mathcal{L}}_{bind}+\lambda \cdot{\mathcal{L}}_{act},$ where $\lambda$ is a hyperparameter balancing the two tasks.

#### Loss functions and optimization

The binding loss$\left({\mathcal{L}}_{bind}\right)$ is calculated using a Binary Cross-Entropy function on the single logit output from the binding head. The activity loss$\left({\mathcal{L}}_{act}\right)$, computed conditionally only for ground-truth binders, uses a Cross-Entropy function on the two-logit output from the activity head. A key limitation in this hierarchical framework is that a binding false negative (FN) in Stage 1—a true binder incorrectly predicted as a nonbinder—prematurely terminates the prediction process, precluding any opportunity for the activity head to evaluate its functional effect. To address this bottleneck, we implemented an error-correcting weight scheme that applies a penalty weight (${w}_{FN}=1.5$) to the activity loss of binding FN samples. This strategy is designed to compensate for the attenuated signal from the gating mechanism for these specific samples; the increased loss forces the activity head to become more sensitive and learn from these low-signal inputs, creating a training dynamic that encourages cooperative error correction. Architectural hyperparameters were determined through sensitivity analysis (Table S4). The model was trained end-to-end by minimizing the composite loss${\mathcal{L}}_{total}$ using the AdamW optimizer with a learning rate of $1\bullet{10}^{-4}$ and a weight decay of $1\bullet{10}^{-5}$ (Table S5).

#### Inference logic

The final prediction (nonbinder, agonist, or antagonist) is determined via a hierarchical inference process. First, the binding probability, ${P}_{bind}$, from the binding head is compared to a primary threshold$\Big({\mathrm{\tau}}_{bind}=$ 0.5 $\Big)$. If ${P}_{bind}<{\mathrm{\tau}}_{bind}$, the compound is classified as a nonbinder. Otherwise, the prediction is determined by the activity head. To mitigate the impact of uncertain binding FNs, this baseline rule is supplemented by a confidence-based rescue logic. This logic overrides the initial nonbinder classification if two conditions are met: (i)${P}_{bind}$ falls within a predefined uncertainty range [0.4, 0.5], and (ii) the activity head’s Softmax probability for either an agonist or antagonist class exceeds a confidence threshold (${\mathrm{\tau}}_{conf}=0.95;$  Table S5. This mechanism allows the model to leverage global allosteric information from the activity head to correct for local uncertainties in the binding head, thereby recovering challenging FN samples. The optimal values for the logic’s hyperparameters were determined via a grid search on the validation set (Fig. S9C).

### Model evaluation and benchmarking

#### Dataset split

The final dataset, used for modeling was curated from GPCRactDB to include 195,989 high-confidence interactions, focusing on three classes: full agonists, antagonists, and nonbinders (Table S1). To rigorously assess generalizability to novel chemotypes, the dataset was partitioned using a stringent scaffold-based splitting strategy [28]. First, Bemis-Murcko scaffolds [29] were generated for all ligands and clustered using the Butina algorithm [30] based on Tanimoto similarity of ECFP4 [31] fingerprints. For the test set allocation, entire ligand clusters were assigned exclusively to the test set until approximately 20% of the data was secured. Crucially, this cluster-based selection was stratified to ensure that class proportions remained representative of the overall dataset while preventing information leakage. Subsequently, the remaining data were randomly divided into training and validation sets at an 80:20 ratio, while preserving overall class proportions.

#### Allosteric complexity benchmark

Given that allosteric modulation is a critical feature for numerous GPCR targets, we designed a benchmark that quantifies each receptor’s allosteric complexity. This complexity was determined by the number of Allosteric Site (AS) residues. For this benchmark, the AS was defined by comparing a receptor’s apo and holo structures; any residue where at least one heavy atom moved more than 4.0 Å between these two states was considered part of the AS after excluding residues that formed the direct binding site.

#### Evaluation metrics

The primary evaluation metric for all the tasks was Balanced Accuracy, chosen for its robustness to class imbalance. The performance was assessed for binary binding prediction, conditional activity prediction, and final three-class prediction (nonbinder, agonist, antagonist). For a comprehensive assessment, we also report Precision, Recall, F1-Score, and Specificity.

### Mechanistic interpretability analysis

To interpret the model’s learned mechanisms, we analyzed the self-attention weights from the Allosteric Propagation Module. The detailed protocols for weight extraction, quantification, and 3D visualization of allosteric pathways are described in the SM6.

## Results

### Sequence-based approaches fail to capture the structural dynamics of activation

Prevailing models for GPCR activity prediction, such as AiGPro and DeepREAL, are limited by their reliance on MSAs. This approach introduces three critical flaws—concerning the input representation, learning signal, and interaction mechanism—that render the models unable to capture essential structural dynamics.

**Figure 3:**
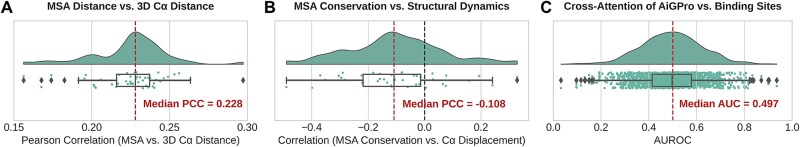
Weak correlation of sequence-based features with GPCR structural dynamics. Analysis of multiple sequence alignments (MSAs) used in sequence-based models. (A) Pearson correlation coefficient (PCC) between MSA residue separation and 3D C$\mathrm{\alpha}$ distance (median PCC = 0.228). (B) Correlation between MSA sequence conservation and C$\mathrm{\alpha}$ displacement during activation (median PCC = −0.108). (C) Area under the receiver operating characteristic (AUROC) for a representative model (AiGPro) cross-attention mechanism identifying binding site residues (median AUROC = 0.497).

First, MSA inputs fail to preserve the receptor’s 3D folding, even with sophisticated BW numbering schemes in AiGPro. Across 94 representative apo GPCR structures, true 3D C$\mathrm{\alpha}$ distances were weakly correlated with 1D MSA separations (median PCC = 0.228; Fig. 3A). This discrepancy highlights an inherent loss of spatial information, evident in the contrast between 3D distance and contact maps (Fig. S2).

Second, the MSA-derived signal of evolutionary conservation negatively correlates with functional activation dynamics. A position’s conservation score, calculated via Shannon Entropy [32], was inversely associated with its mean C$\mathrm{\alpha}$ displacement upon activation (median PCC = −0.108; Fig. 3B). This indicates that the models are biased to prioritize structurally static residues, systematically ignoring the dynamic regions essential for function.

Third, cross-attention mechanisms, which are designed to learn protein-ligand interactions, do not reliably identify physical binding sites. To permit mechanistic analysis, we retrained an AiGPro model and evaluated its attention patterns on a benchmark of 1940 GPCR-ligand pairs. The model’s ability to distinguish true binding residues yielded an AUROC of 0.497—statistically equivalent to random chance (Fig. 3C). This result indicates that the model overfits spurious sequence correlations rather than learning generalizable physical interactions.

### Structural geometry encodes a deterministic signal for G-protein-coupled receptor activation

To first establish whether resolved 3D structures contain sufficient information to distinguish functional states, we performed an exploratory analysis using an interpretable classification and regression tree (CART) [33] model. The model was fit to the entire dataset of 617 known GPCR structures not to test generalizability, but to probe the information content inherent in the geometry itself. The features included intra-receptor C$\mathrm{\alpha}$ distances and per-residue C$\mathrm{\alpha}$ displacements from a reference apo state. A single highest-resolution experimental apo structure was used as the reference where available (see SM5). For receptors lacking a high-quality experimental apo structure, the AlphaFold2 predicted model was used as a substitute (Fig. S3).

**Figure 4:**
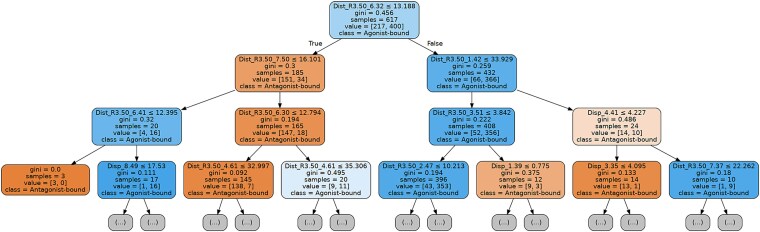
A decision tree reveals the deterministic structural signal for GPCR activity. A classification and regression tree (CART) model was fit to the entire dataset of 617 resolved GPCR structures to probe the information content inherent in 3D-derived features.

The resulting decision tree could perfectly partition the dataset into agonist-bound and antagonist-bound states using only a few structural parameters (Fig. 4), demonstrating that these features contain a deterministic signal. Notably, the root node alone—a distance threshold between TM3 and TM6 (Dist_R3.50_6.32 ≤ 13.188 Å)—separated 83.8% of the structures (517/617). Subsequent splits also prioritized distance-based (final state) features over displacement-based (process) features. This analysis confirms that a resolved 3D structure effectively contains the answer to its conformational state.

Furthermore, ligand-induced displacements form a conserved, MoA-dependent profile. A BW-aligned mean C$\mathrm{\alpha}$ displacement analysis revealed that agonists elicit larger conformational changes in regions critical for G-protein coupling, namely, the intracellular ends of TM5 and TM6 (Fig. S4). In contrast, antagonists induced pronounced shifts in the adjacent regions of extracellular loop 3. Thus, while a resolved 3D structure contains sufficient information to determine activity, predicting this conformational outcome for uncharacterized GPCR-ligand pairs remains a fundamental allosteric problem.

### GPCRactDB: a comprehensive data foundation for ligand-induced activity prediction

**Figure 5:**
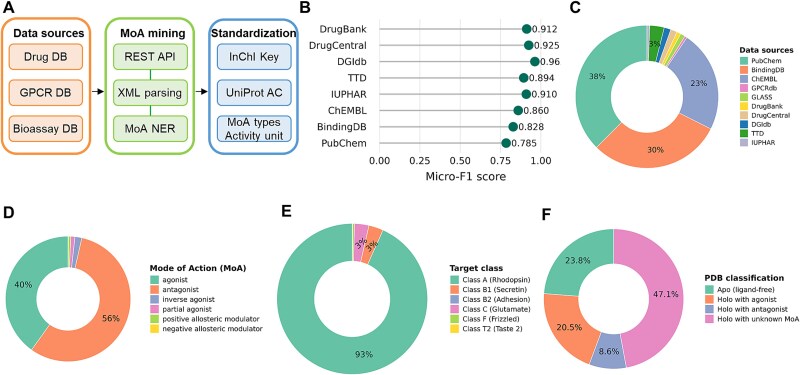
Construction and characterization of the GPCRactDB. (A) The data integration pipeline for aggregating and standardizing functional data. (B) Validation of the mode of action (MoA) annotation pipeline against the GPCRdb gold-standard set. (C) Contribution of public data repositories to the final dataset. (D) Distribution of curated MoA labels. (E) Coverage of major GPCR classes. (F) Conformational and ligand-binding states of functionally annotated human GPCRs.

To address the fragmented nature of GPCR functional data, we constructed GPCRactDB, a new large-scale database that unifies functional and structural information. The database was built via a systematic pipeline (Fig. 5A; Fig. S5). A high-fidelity MoA annotation pipeline ensures data quality, achieving an average micro-F1 score of 0.885 when validated against a gold-standard set of approved drugs (Fig. 5B). Applying this validated pipeline, we then integrated these sources to construct the final database, which is primarily composed of data from PubChem, BindingDB, and ChEMBL (Fig. 5C). This process yielded GPCRactDB, a comprehensive resource covering six distinct MoA categories and containing 202,925 unique ligand-GPCR interactions across 295 human GPCRs (Fig. S6; Supplementary Data 1). The resulting dataset is functionally diverse, covering all major MoA and GPCR classes (Fig. 5D and E). This represents a substantial advance in scale and specificity over existing resources such as MolData [34] (Fig. S7). Finally, these functional annotations were mapped to 1602 experimentally resolved GPCR structures, classifying the human GPCR structural landscape into distinct functional states (Fig. 5F). This integrated database provides the essential foundation for all subsequent analyses.

### Optimizing the GPCRact architecture: a bottom-up validation

The final architecture of GPCRact was established through a series of data-driven, bottom-up validation processes. We began by optimizing components for local ligand binding prediction, then integrated them into the end-to-end framework to optimize the full activity prediction task.

The design of GPCRact begins with the protein input representation. While computationally efficient, C$\mathrm{\alpha}$-only graphs inherently ignore the sidechains critical for ligand interaction. We therefore benchmarked different atomistic graph schemes on a binding prediction task to find an optimal balance of biophysical detail and efficiency, using a test set with a stringent ligand scaffold-based split. Our results confirm that including specific sidechain information is essential for accurate binding prediction (Table 1). A C$\mathrm{\alpha}$-only baseline yielded a BAcc of 0.811, whereas a computationally intensive all-heavy-atom graph improved the performance to 0.852. The proposed hybrid representation, C$\mathrm{\alpha}$ + key functional atoms, achieved the highest performance (BAcc 0.890), establishing its superior balance of predictive power and efficiency.

**Table 1:** Performance of different protein graph representations on the binding prediction task.

Representation	Precision	Recall	F1 Score	Specificity	BAcc
C$\mathrm{\alpha}$-only (Baseline)	0.962	0.874	0.916	0.747	0.811
All heavy-atom	0.969	0.892	0.929	0.811	0.852
C$\mathrm{\alpha}$+ key functional atoms	0.978	0.907	0.941	0.873	0.890

Next, we optimized the protein-ligand interaction module through an ablation study of its cross-attention mechanism (Table 2). A simple feature concatenation baseline performed moderately (BAcc 0.838), and unidirectional attention offered no significant improvement. However, a bidirectional cross-attention mechanism, which allows for mutual feature updates between the protein and ligand, yielded the best performance (BAcc 0.865). This gain was driven by a substantial increase in Specificity (0.896 versus 0.844), confirming the importance of modeling reciprocal interactions.

**Table 2:** Performance of different interaction modeling strategies on the binding prediction task.

Interaction strategy	Description	Precision	Specificity	BAcc
None (Baseline)	Simple concatenation of P & L embeddings	0.963	0.844	0.838
$\mathrm{P}\to \mathrm{L}$ (Unidirectional)	Ligand queries information from Protein	0.959	0.834	0.813
$\mathrm{L}\to \mathrm{P}$ (Unidirectional)	Protein queries information from Ligand	0.963	0.846	0.832
Bidirectional	Mutual information exchange between P & L	0.934	0.896	0.865

Having established the optimal modules for binding prediction, we finalized the end-to-end architecture for the three-class activity prediction task. We performed a stepwise ablation study to validate the final design, comparing distinct information flow strategies and core components for allosteric propagation (Table 3). In this hierarchy, the superior model from each part served as the baseline for the next, demonstrating a clear trajectory of performance improvement.

**Table 3:** Ablation studies validating the GPCRact architecture and core components for three-class activity prediction.

Model configuration	BAcc	Non-binder recall	Antagonist recall	Agonist recall
**Part A: Framework architecture**				
1a. Unified single-head	0.713	0.654	0.768	0.717
1b. Shared-representation multitask	0.733	0.682	0.781	0.736
1c. Hierarchical (Decoupled)	0.766	0.729	0.805	0.764
**Part B: Allosteric propagation layers (Baseline: Model 1c)**				
2a. Hierarchical w/ EGNN-only	0.766	0.729	0.805	0.764
2b. Hierarchical w/ EGNN + Self-attention	0.787	0.758	0.819	0.784
**Part C: Inter-module signal transfer (Baseline: Model 2b)**				
3a. Direct signal transfer	0.787	0.758	0.819	0.784
3b. Gated signal transfer	0.814	0.791	0.842	0.809
**Part D: Inference logic strategy (Baseline: Model 3b)**				
4a. Standard inference (w/o Rescue)	0.814	0.791	0.842	0.809
4b. Confidence-based rescue (Optimized)	0.819	0.757	0.873	0.829

First, we evaluated three architectural frameworks to determine the optimal information flow for activity prediction (Part A). A baseline unified single-head model (1a), which attempts to predict all three classes from a single feature vector, showed moderate performance (BAcc 0.713). Separating the tasks in a shared-representation multitask architecture (1b), where both binding and activity heads use the same final propagated features, improved performance to a BAcc of 0.733. However, our proposed hierarchical (decoupled) architecture (1c) proved optimal, achieving the highest BAcc of 0.766. This design explicitly decouples the information flow: the binding head utilizes local features prior to allosteric propagation, while the activity head uses global features from after. The resulting +3.3%p BAcc gain over the shared model (1b) confirms our hypothesis that specializing each task with the most relevant information is the superior strategy. This is further evidenced by the marked increase in nonbinder recall (0.729 versus 0.682), which underscores the model’s improved ability to identify nonbinders using dedicated local information.

Crucially, to rigorously verify that this hierarchical design effectively decouples binding from activity prediction without degradation, we conducted a decomposed performance evaluation (Table S6). The hierarchical model outperformed the shared-representation baseline in both the binding task and the conditional activity task. Furthermore, consistency analysis confirms that the model rationally treats binding as a functional prerequisite; activity predictions are highly accurate for correctly identified binders but collapse to near-random performance when binding is incorrectly predicted (Fig. S8). Subsequently, using the hierarchical model as the new baseline, we evaluated the allosteric propagation module (Part B). Integrating a global self-attention mechanism alongside local EGNN layers (2b) improved the BAcc by +2.1%p to 0.787. This confirms that a hybrid approach—where EGNN captures local geometric changes and self-attention models long-range communication—is essential for modeling allostery.

We then refined the signal transfer mechanism between the binding and activity modules (Part C). Introducing a gated signal transfer mechanism (3b) provided the most substantial performance boost, increasing the BAcc by +2.7%p to 0.814. This improvement was underpinned by a balanced increase in recall across all classes, with nonbinder recall notably rising from 0.758 to 0.791, confirming that the gate facilitates a cooperative error-correction dynamic.

As a final optimization step, we implemented a confidence-based rescue mechanism during inference (Part D). Our calibration analysis revealed that the activity head is highly reliable for conditional prediction (ECE = 0.002; Fig. S9A and B), serving as a trustworthy proxy to recover false negatives from the binding head. Applying this logic with statistically optimized thresholds (Fig. S9C) increased the final BAcc to 0.819. Crucially, this refinement recovered a significant portion of functional ligands (antagonist recall +3.1%p, agonist recall +2.0%p), with a controlled trade-off in nonbinder recall (−3.4%p).

In summary, this sequential validation demonstrates a clear trajectory of performance improvement, with the BAcc progressively increasing from the baseline hierarchical model (1c, 0.766) through the addition of self-attention (2b, 0.787) and the gated architecture (3b, 0.814) to the final inference strategy (4b, 0.819).

### GPCRact demonstrates robust performance on allosterically complex targets

**Figure 6:**
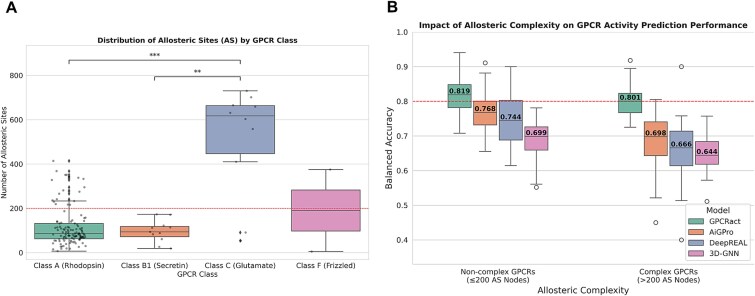
Impact of allosteric complexity on GPCR activity prediction. (A) Distribution of allosteric site (AS) counts across GPCR classes. Receptors with >200 AS residues (red dashed line) are defined as ‘allosterically complex.’ (B) Comparison of balanced accuracy for GPCRact, sequence-based SOTA models (AiGPro, DeepREAL), and a 3D-GNN baseline on benchmark sets stratified by allosteric complexity.

To evaluate the capacity of GPCRact to model allosteric communication, we benchmarked its performance across targets stratified by structural complexity, which was quantified by the number of Allosteric Site (AS) residues. An analysis of known GPCR structures confirmed that allosteric complexity varies significantly across classes, with Class C receptors possessing a much larger AS network (Fig. 6A). We therefore designated receptors with over 200 AS residues as ‘allosterically complex,’ defining a challenging subset where we hypothesized that sequence-based models would underperform.

Across both subsets (allosterically complex and noncomplex), GPCRact consistently outperformed state-of-the-art sequence-based models, with its advantage becoming most pronounced for complex targets (Fig. 6B). On noncomplex GPCRs, GPCRact already achieved a superior median BAcc of 0.819. This performance gap widened dramatically on the allosterically complex subset, where the accuracies of AiGPro and DeepREAL decreased sharply by 7.0%p and 7.8%p, respectively. In stark contrast, GPCRact’s performance remained highly robust, declining by only 1.8%p to maintain a high BAcc of 0.801. This robustness for complex targets, where sequence-based models systematically underperform, validates GPCRact’s foundational design of leveraging 3D structural information to capture the mechanisms of allosteric communication.

To distinguish the contributions of input modality versus architectural design, we additionally evaluated a simplified 3D-GNN baseline model. This baseline utilizes the same protein and ligand graphs as GPCRact but lacks the hierarchical architecture and attention-based propagation modules. While this model exhibited greater stability than sequence-based models—declining by only 5.5%p (0.699–0.644) on complex targets—its absolute accuracy remained uncompetitive and substantially lower than that of GPCRact across both subsets. These results indicate that 3D modality alone is insufficient for high performance, and that GPCRact’s architectural design plays a key role in capturing the biophysical determinants of activity.

### Mechanistic insight: GPCRact learns biologically relevant allosteric pathways

To validate the internal mechanisms of GPCRact and ensure interpretability beyond a black box approach, we analyzed the self-attention weights from the final global integration layer of the Allosteric Propagation Module. We calculated a normalized importance score for each residue and grouped these scores by known functional motifs within several allosterically complex Class A families.

**Figure 7:**
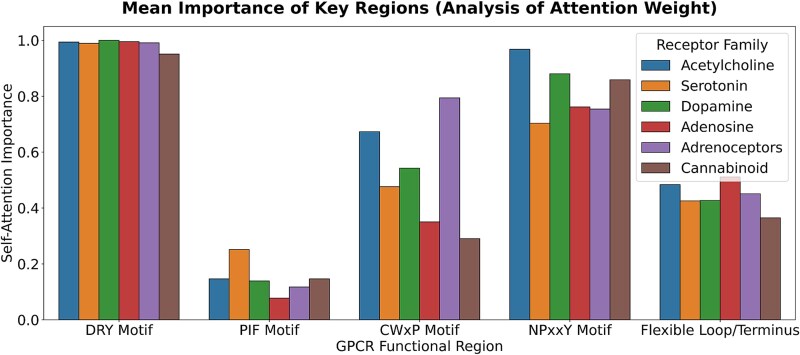
Mean self-attention importance of key GPCR functional motifs. Bar chart comparing the mean importance of self-attention for canonical functional regions across several class A receptor families. The importance score for each residue, derived from the model’s self-attention weights, is normalized to a rank percentile (0.0–1.0) for comparison.

The analysis revealed that the model’s learned attention quantitatively corresponds to the biological importance of key GPCR activation motifs (Fig. 7). Across all the tested families, the model consistently assigned the highest importance to the highly conserved DRY motif (R3.50), reflecting its universal role as a critical activation switch [25]. Meanwhile, the NPxxY (Y7.53) and CWxP (W6.48) motifs exhibited high but variable importance, reflecting family-specific regulatory differences. For example, the NPxxY motif has received significantly more attention in acetylcholine and dopamine receptors, consistent with its prominent role in$\mathrm{\beta}$-arrestin-mediated signaling [35]. In contrast, the PIF motif consistently received the lowest attention scores across all families. Functionally less critical regions, including flexible loops and termini, also received low scores, collectively demonstrating the model’s ability to distinguish core activation machinery from nonintegral regions.

To move beyond correlation and validate the causal nature of these learned weights, we performed an *in silico* point mutation study on the canonical R3.50 activation switch (SM 6.4). We introduced the well-characterized, function-abolishing R3.50A mutation [36, 37] into the ${\mathrm{\beta}}_2$-adrenergic receptor (ADRB2). As shown in Fig. S10, this single-residue mutation—which disrupts the physical allosteric pathway—caused the model’s agonist probability to collapse (mean P(Agonist): 0.916–0.457). Crucially, this effect was specific to the functional prediction: the Stage 1 binding probability remained largely unaffected (decreasing by only 0.131). This aligns with the mutation’s distal location relative to the binding site and confirms that the model has learned to strictly decouple the binding event from the functional outcome based on structural integrity.

Complementing this protein-centric view, we analyzed the ligand-level features driving classification via cross-attention analysis on ADRB2 (Table S10). This revealed that the model learns chemically intuitive pharmacophores, assigning significantly higher importance to specific atomic features such as ‘Sulfur in agonists’ and ‘Chlorine in antagonists,’ thereby validating its holistic understanding of the physicochemical determinants of activation [38].

Finally, to contextualize our model’s learned functional map against classical biophysical frameworks, we conducted a comparative analysis with Perturbation Response Scanning (PRS) [14] (SM 6.6). First, the mean PRS sensor profile across structural states (apo, agonist-, antagonist-bound) reproduced the well-established characteristic: the highest importance was assigned to physically flexible regions, such as loops (Fig. S11). This confirms that PRS accurately captures intrinsic structural flexibility. Second, we evaluated whether a differential, state-dependent PRS profile (agonist- versus antagonist-bound) could reveal a discriminative functional signature. This analysis did not exhibit a consistent pattern at the motif level (e.g. DRY did not show stable directional changes). This result reinforces a fundamental distinction in methodological scope: whereas PRS maps intrinsic physical dynamics, GPCRact is explicitly designed to learn a ligand-conditioned functional map. As shown in Fig. S12, our model learns a dynamic attention pattern that systematically assigns differential importance to canonical motifs based on the ligand’s specific MoA.

**Figure 8:**
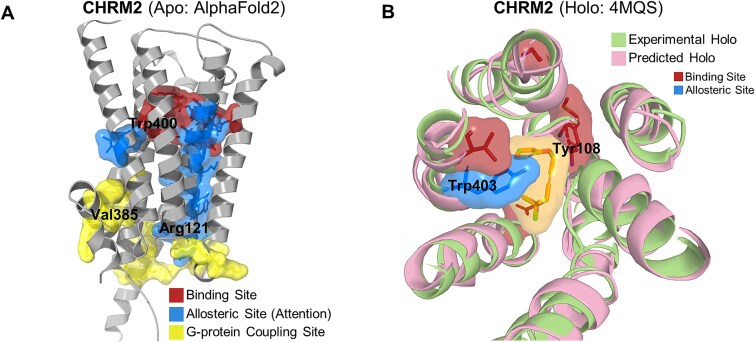
Case study on the M2 muscarinic acetylcholine receptor (CHRM2). (A) Visualization of the predicted allosteric pathway on the receptor’s apo structure. Residues with the highest self-attention scores (blue) connect the orthosteric binding site (red) and the G-protein coupling site (yellow). (B) Comparison of the experimental active holo state (green) with the model-inferred conformation from GPCRact (pink), which was derived from the apo structure as input.

To provide a direct visual demonstration of these validated principles, we performed a case study on the M2 muscarinic acetylcholine receptor (CHRM2). Mapping the residues with the highest self-attention scores onto the receptor apo structure [39] revealed a physically contiguous pathway of high-importance residues connecting the orthosteric binding site to the distal G-protein coupling site (Fig. 8A).

We then investigated whether the model’s internal geometric reasoning corresponded to real-world conformational changes by inspecting the final coordinates from its equivariant layers. Remarkably, when processing only the apo structure, this internal representation—learned solely for the classification task—showed structural similarity to the experimentally determined holo conformation [40] (Fig. 8B). The alignment of key residues within both the binding site and the learned allosteric pathway was particularly close. This observation suggests that to correctly classify activity, GPCRact has learned to approximate the geometric transformation from an input apo to an active-like conformation.

## Discussion

In this study, we developed GPCRact, a hierarchical framework that predicts ligand-induced GPCR activity by modeling allosteric communication. This structure-aware approach directly resolves a central flaw of sequence-based paradigms: their failure to account for the three-dimensional geometry that governs activation dynamics. Consequently, underpinned by our comprehensive GPCRactDB, the framework delivers on two critical fronts: it achieves robust accuracy on allosterically complex receptors while enhancing interpretability by validating its predictions against known biological pathways.

Our framework is built upon the premise that the final 3D structure of a GPCR complex deterministically encodes its functional state; however, the central challenge is predicting this conformational outcome for unresolved ligand-receptor pairs. GPCRact addresses this by employing a modular architecture designed to learn the mapping from an initial set of ligand and receptor structures to a final functional state. This architecture integrates EGNN and attention mechanisms to capture the biophysical principles connecting ligand binding to the eventual conformational outcome. The framework’s strength derives from its specialization: cross-attention is dedicated to the initial binding event, while a distinct propagation module combines local geometric updates with global information flow to capture long-range allosteric communication. This approach marks a shift from correlational sequence analysis to structure-based causality. It is distinct from sequence-based black box models, which lack 3D physical mechanisms, and classical biophysical frameworks. While powerful, the latter analytical methods (e.g. PRS, ENM) profile state-dependent intrinsic dynamics but are not designed to predict functional outcomes for novel ligands. Recognizing the computational complexity of modeling full-protein dynamics, our framework is strategically designed to learn the minimal sufficient information from key allosteric and binding site regions to achieve its predictive goal. This design enables the activation process to be deconstructed for mechanistic interpretation.

Beyond predictive accuracy, the ultimate value of GPCRact lies in its mechanistic fidelity. The framework’s significance is validated not by the discovery of novel pathways, but by its ability to independently deduce the functional hierarchy of canonical activation sites, such as the DRY motif, from 3D structural data alone. This recapitulation serves as a critical validation of the model’s internal logic, confirming that it operates on biologically plausible principles rather than spurious correlations. Our mechanistic validation confirms these principles are not just correlational but causal, as the model’s predictions are dependent on the structural integrity of these distal pathways. This mechanistic understanding is further illustrated in our case study on the M2 receptor, where the model’s internal representation, learned solely from an apo input, captured key structural features of the final holo state. This observation reinforces that the model operates on biologically relevant principles rather than simply classifying labels, transforming it into a tool for generating testable hypotheses. The principles of this approach are broadly applicable to other allosteric systems, and GPCRactDB provides an essential foundational resource to catalyze further innovation within the drug discovery community.

Despite its robust performance and mechanistically grounded design, the GPCRact framework has several key limitations. First, the model relies on simplified input representations to ensure computational efficiency. Specifically, the use of static apo structures does not fully capture the intrinsic flexibility of proteins. Simultaneously, the utilization of a single generated ligand conformer may overlook the conformational diversity of flexible ligands. However, our extensive sensitivity analysis (Supplementary Results; SR2) mitigates this concern by demonstrating that predictions remain stable across conformational ensembles, suggesting the model learns robust pharmacophoric features rather than overfitting to specific coordinates. It is important to note that while the framework’s internal coordinate updates can result in a final conformation that resembles the holo state in specific cases, it is not a generative method designed to produce novel holo structures like AlphaFold3 [4], nor does it explicitly simulate the atomistic trajectory of activation in the manner of molecular dynamics (MD) simulations [41]. Second, the dataset reflects the inherent bias of public GPCR repositories, with Class A receptors constituting approximately 93% of GPCRactDB. Although stratified analysis (Table S7) indicates stable performance across Classes B1, C, and F, this imbalance inevitably constrains generalization to rare structural motifs found in underrepresented classes. Third, a fundamental challenge for structure-based models is generalizing to entirely unseen receptor topologies. While GPCRact demonstrates robust generalization to novel chemotypes under the global scaffold-split setting, our multilevel out-of-distribution (OOD) analysis reveals the model’s structural boundaries. In the Leave-One-Receptor-Out analysis (Table S8), the model maintained predictive power (BAcc 0.63–0.77) on unseen targets by leveraging family-conserved structural motifs. However, the Leave-One-Family-Out analysis (Table S9) showed a further reduction in accuracy when applying to *de novo* receptor families. This performance gradient indicates that the Allosteric Propagation Module encounters a latent-space distribution shift when the geometric manifold of the target differs significantly from those seen during training. Nevertheless, the fact that performance consistently exceeds the random baseline—even in rigorous OOD settings—confirms that the model captures conserved biophysical principles of activation rather than relying solely on simple pattern matching. Finally, the current scope is restricted to orthosteric agonists and antagonists, not yet accounting for complex mechanisms such as inverse agonism or allosteric modulation.

However, these limitations inform several promising avenues for extending the framework’s scope and impact. Technically, integrating MD data would better capture protein flexibility, while coupling the framework with generative models could enable the design of ligands that actively sculpt the receptor’s conformational landscape. Biologically, its scope can be deepened to address nuanced questions such as predicting G-protein- versus $\mathrm{\beta}$-arrestin-biased signaling and explicitly incorporating the effects of allosteric modulators by building upon the foundational allosteric framework established here. Most significantly, the framework’s logic can be effectively leveraged for drug discovery. As demonstrated in our two-stage simulation (SR4), this hierarchical approach enables an efficient strategy for not only identifying hits but also prioritizing them by their functional MoA. Furthermore, the principles of this structure-based approach can be extended to other critical target classes where activation dynamics are essential, such as nuclear receptors, to broaden its impact.

GPCRact unites robust predictive accuracy for allosterically complex targets with deep mechanistic interpretability, resolving a core dilemma in computational drug discovery. This approach marks a paradigm shift, moving beyond the correlational limitations of sequence-based analysis and the predictive limitations of classical biophysics, advancing towards mechanistically grounded model of the causal, biophysical principles governing protein activation. As such, it provides a blueprint for the next generation of predictive tools in structural biology and opens a more efficient and rational era of structure-guided drug discovery.

**Key Points**

- GPCRact introduces a structure-aware framework that models allosteric communication to predict ligand-induced GPCR activity, overcoming a fundamental limitation of traditional sequence-based methods.
- Its dual attention architecture on an equivariant graph network hierarchically models the biophysical process of activation: cross-attention for initial ligand binding and self-attention for signal propagation to distal functional sites.
- The model achieves state-of-the-art accuracy, with particularly robust performance on allosterically complex receptors, a domain where sequence-based models systematically underperform.
- The model’s learned attention weights are consistent with known allosteric pathways, validating its internal logic and establishing a framework for generating plausible hypotheses for uncharacterized GPCR-ligand interactions.

## Supplementary Material

Son_BIB_Supplementary_Publish_bbaf719_Final

Son_BIB_SupplementaryData1_bbaf719

## Data Availability

All raw data used in this study were obtained from the ten public repositories listed in the Supplementary Methods (SM1), with specific versions and access dates provided. Structural data were sourced from the RCSB PDB and the AlphaFold Protein Structure Database. The complete code for data retrieval, curation, model training, and figure generation is publicly available on GitHub at https://github.com/hyojin0912/HJ-GPCRact.
