# Supplementary material for: GPCRact: a hierarchical framework for predicting ligand-induced GPCR activity via allosteric communication modeling
Source: Brief Bioinform. 2026 Jan 15;27(1):bbaf719. doi: 10.1093/bib/bbaf719 (PMC12805254; doi:10.1093/bib/bbaf719)
Supplement: Son_BIB_Supplementary_Publish_bbaf719_Final [file son_bib_supplementary_publish_bbaf719_final.docx]

**Supplementary information for**

**GPCRact: a hierarchical framework for predicting ligand-induced GPCR activity via allosteric communication modeling**

**Hyojin Son^1^ and Gwan-Su Yi^1*^**

1 Department of Bio and Brain Engineering, Korea Advanced Institute of Science and Technology (KAIST), Daejeon, Republic of Korea

**Table of Contents**

**Supplementary Methods**

[SM1](#_SM1._Detailed_GPCRactDB). Detailed GPCRactDB curation pipeline

[SM2](#_SM2._Detailed_definition_1). Detailed definition of graph components

[SM3](#_SM3._Baseline_model). Baseline model implementation and training

[SM4](#_SM4._GPCRact_hyperparameter). GPCRact model optimization and performance validation

[SM5](#_SM5._Classification_and_1). Classification and Regression Tree (CART) model for structural signal analysis

[SM6](#_SM6._Mechanistic_interpretability). Mechanistic interpretability analysis protocol

[SM7](#_SM7._Validation_of_1). Validation of AlphaFold2 structures as proxies for apo states

[SM8](#_SM8._Proof-of-concept_virtual). Proof-of-concept virtual screening and lead prioritization simulation

# [SM9](#_SM9._Implementation_details). Implementation details and computational environment

**Supplementary Tables**

[Table S1](#_Table_S1._Distribution). Distribution of the three-class modeling dataset.

[Table S](#_Table_S2._Detailed)2. Detailed breakdown of feature categories for different protein graph representations.

[Table S](#_Table_S2_Definition)3. Definition of key functional sidechain atoms.

[Table S4.](#_Table_S4._Hyperparameter) Hyperparameter sensitivity and efficiency analysis.

[Table S](#_Table_S3_Final)5. Final hyperparameters for the GPCRact model.

[Table S6](#_Table_S6._Decomposed). Decomposed performance of architecture variants on binding and conditional activity tasks.

[Table S7](#_Table_S7._Stratified). Stratified performance evaluation across GPCR classes.

[Table S8](#_Table_S8._Generalization). Generalization performance on unseen receptors (LORO).

[Table S9](#_Table_S9._Generalization). Generalization performance on unseen receptor families (LOFO).

[Table S10](#_Table_S10._Differential). Differential cross-attention analysis of ligand pharmacophores for ADRB2.

**Supplementary Results**

[SR1](#_SR1._Logical_consistency). Logical consistency analysis of hierarchical predictions

[SR2](#_SR2._Sensitivity_analysis). Sensitivity analysis of ligand conformational variability

[SR3](#_SR3._Comparative_analysis). Comparative analysis of allosteric maps and MoA-specific attention

[SR4](#_SR4._Case_study:). Case study: Utility in virtual screening and lead prioritization

**Supplementary Figures**

[Figure S1](#_Figure_S1._Validation). Validation of functional site consistency.

[Figure S](#_Figure_S1._Comparison)2. Comparison of 3D raw distance maps and 2D contact maps.

[Figure S](#_Figure_S3._Validation)3. Validation of AlphaFold2 (AF2) structures as apo state proxies.

[Figure S](#_Figure_S4._BW-aligned)4. BW-aligned mean C$\alpha$displacement profiles for agonist- vs. antagonist-bound GPCRs.

[Figure S](#_Figure_S5._Overall)5. Overall workflow for the construction of the GPCRactDB.

[Figure S](#_Figure_S4._Detailed)6. Hierarchical text mining pipeline for PubChem BioAssay curation.

[Figure S](#_Figure_S5._Quantitative)7. Quantitative scale comparison of GPCRactDB and MolData database.

[Figure S8](#_Figure_S8._Logical). Logical consistency of activity predictions conditional on binding outcomes.

[Figure S9](#_Figure_S9._Statistical). Statistical validation and hyperparameter optimization of the confidence-based rescue logic.

[Figure S10](#_Figure_S10._Causal). Causal validation of GPCRact via in silico point mutation of the R3.50 activation switch.

[Figure S11](#_Figure_S11._Mean). Mean PRS sensor profile of key functional regions.

[Figure S12](#_Figure_S12._Ligand-dependent). Ligand-dependent (MoA-specific) modulation of attention patterns.

**Supplementary Methods**

# **SM1. Detailed GPCRactDB curation pipeline**

To establish a comprehensive and unified data foundation for this study, we constructed GPCRactDB by systematically integrating and curating information from ten public repositories. The overall workflow is illustrated in Figure S5. The curation process consisted of four main stages: (i) data extraction, (ii) mode of action (MoA) annotation and label sourcing, (iii) data standardization, and (iv) filtration for final model dataset construction.

**SM1.1. Data sources and extraction** We aggregated data from three distinct categories of databases. First, data for approved drugs were collected via REST APIs from DrugBank (v5.1.12) [1], DrugCentral (2023-11-01) [2], DGIdb (v5.0.7) [3], and the Therapeutic Target Database (TTD, 2024-01-10) [4]. Second, GPCR-specific functional and structural data were sourced from curated tables in GPCRdb (2024-07-16) [5] and the GLASS database (v19-02-23) [6]. Third, large-scale bioassay data were extracted from PubChem BioAssay (2024-11-01) [7], ChEMBL (v34) [8], BindingDB (2024-11) [9], and the IUPHAR/BPS Guide to PHARMACOLOGY (2024-03) [10]. For unstructured text from bioassay databases, particularly PubChem (from an initial set of 35,251 Assay IDs), a dictionary-based named entity recognition (NER) pipeline was developed to parse assay descriptions and extract MoA-related entities. This initial processing yielded 21,555 relevant assays for further annotation.

**SM1.2. MoA annotation** Extracted data were systematically annotated into one of six primary MoA classes: agonist, antagonist, partial agonist, inverse agonist, positive allosteric modulator (PAM), and negative allosteric modulator (NAM). This structured annotation was applied across all data sources to create a harmonized functional dataset. Specifically, functional labels (e.g., agonist, antagonist) were directly sourced from curated database fields (DrugBank, GPCRdb) or extracted from bioassay descriptions using the MoA-NER pipeline (Fig. S6). In contrast, non-binder labels were exclusively defined based on explicit binding assays (e.g., radioligand displacement, SPR, ITC) where no interaction was observed below the activity threshold.

**SM1.3. Data standardization and conflict resolution** To ensure data consistency, all small molecules and proteins were mapped to standardized identifiers, specifically InChIKeys and UniProt Accession numbers [11], respectively. Dose-response values (e.g., ${IC}_{50}, {EC}_{50}, K_{i}, K_{d}$) were normalized to a standard unit of$\mu M$, and a cutoff of 10$\mu M$was applied to binarize activity. In cases where a single ligand-GPCR pair had conflicting MoA annotations from different sources, a majority voting scheme was applied. Any remaining conflicts were subsequently handled through manual curation according to predefined rules, such as excluding pairs labeled as both agonist and antagonist, or prioritizing a partial agonist annotation over a full agonist label. This process resulted in a final curated set of 202,925 unique ligand-GPCR interactions across 295 human GPCRs.

**SM1.4. Dataset filtration for three-class modeling** The comprehensive GPCRactDB provides a broad functional landscape across six MoA categories. For the predictive modeling task, however, the dataset was filtered to retain only the three mechanistically coherent and pharmacologically dominant classes: full agonists, antagonists, and non-binders. This filtration was motivated by three key considerations. First, the GPCRact architecture is specifically designed to model the biophysical consequences of orthosteric binding (Stage 1) leading to allosteric signal propagation (Stage 2). Including ligand types whose primary mechanism relies on non-orthosteric initiation would be mechanistically inconsistent with this causal structure. Second, several ligand classes exhibit mechanisms that do not align with the orthosteric efficacy axis. For instance, partial agonists produce submaximal activation even at full receptor occupancy, inverse agonists reduce constitutive signaling, and allosteric modulators alter receptor responses in a context-dependent manner. Incorporating these heterogeneous mechanisms into a unified label space would confound the model’s ability to learn the orthosteric signaling patterns that it is designed to capture. Third, full agonists and antagonists constitute approximately 95% of approved GPCR-targeting therapeutics [5], representing the most critical classes for translational drug discovery. Based on these rationales, all entries labeled as partial agonist, inverse agonist, PAM, or NAM were excluded. After filtration and verification of valid SMILES representations, the final modeling dataset contained 195,989 ligand–GPCR interactions. The detailed class distribution and scaffold-based partitioning are reported in Table S1.

# **Table S1**. Distribution of the three-class modeling dataset.

| **Class** | **Training** | **Validation** | **Test** | **Total** |
| --- | --- | --- | --- | --- |
| Antagonist | 56,707 | 14,248 | 17,779 | 88,734 |
| Agonist | 40,508 | 10,056 | 11,432 | 61,996 |
| Non-binder | 28,165 | 7,042 | 10,052 | 45,259 |
| **Total** | **125,380** | **31,346** | **39,263** | **195,989** |

#

# **SM2. Detailed definition of graph components**

This section provides the detailed protocols for constructing the protein and ligand graphs used as input for GPCRact.

**SM2.1. Protein graph construction** The protein graph was constructed as a functionally critical subgraph covering both binding and allosteric sites. To ensure a robust and biologically representative definition, these sites were defined based on a consensus of all available structural evidence, a process detailed below.

**Node definition (functional sites)**: The graph nodes consist of all heavy atoms from the union of the consensus Binding Site (cBS) and the consensus Allosteric Site (cAS).

- The cBS for a given receptor was defined by aggregating all residues with any heavy atom within 4.0$Å$of a bound ligand. This analysis was performed across all available experimental holo structures for that receptor.
- The cAS was defined as the top 100 non-BS residues exhibiting the most significant conformational change. To calculate this, all available holo structures for a given receptor were superimposed onto a single, representative apo structure based on their transmembrane (TM) domains. The mean C$\alpha$displacement was then calculated for each residue, and the top 100 were selected.

**Node features**: Each heavy atom in the protein graph is represented by a 37-dimensional feature vector. This vector combines residue-level information, set membership (cBS or cAS), and atom-level properties. A detailed breakdown is provided in Table S2.

# **Table S2**. Detailed breakdown of feature categories for different protein graph representations.

| **Feature category** | **C**$\boldsymbol{\alpha}$**-only** | **All heavy-atom** | **C**$\boldsymbol{\alpha}$**+ functional atoms** |
| --- | --- | --- | --- |
| Amino acid type (Residue) | ✓ | ✓ | ✓ |
| Set membership (is BS/AS) | ✓ | ✓ | ✓ |
| Atom-level properties | | | |
| ┝ Atom type & coordinates | N/A | ✓ | ✓ |
| ┝ RDKit chemical features | N/A | ✓ | ✓ |
| ┝ Node role (C$\alpha$vs. sidechain) | N/A | N/A | ✓ |
| **Total dimension** | **23-dim** | **35-dim** | **37-dim** |

**Edge construction**: Edges in the protein graph were defined using a k-Nearest Neighbors (k-NN) approach to ensure a uniform graph density for stable model training. The value of k=64 was selected based on an empirical analysis comparing two validation steps: (i) a comparison against a fixed-radius cutoff method (8.0$Å$), and (ii) a systemic sensitivity analysis across varying neighbor counts ($k\in\{32, 64, 128, 256\})$. While the radius-cutoff method created graphs of highly variable density, leading to prohibitive GPU memory usage for larger receptors, the k-NN approach provided a superior balance. Specifically, k=64 achieves the optimal trade-off, maximizing predictive performance while preventing the over-smoothing and gradient instability observed at higher densities (e.g., k=256).

**Definition of key functional atoms**: For the final graph representation, in addition to the C$\alpha$atom, key functional atoms from each amino acid’s sidechain were included to better capture specific chemical properties. The selection of these atoms, as detailed in Table S3, was based on their role in hydrogen bonding, charge, aromaticity, and steric properties.

# **Table S3**. Definition of key functional sidechain atoms.

| **Category** | **Amino Acid** | **Key Functional Atoms** |
| --- | --- | --- |
| Aliphatic | Alanine (ALA) | CB |
|  | Valine (VAL) | CG1, CG2 |
|  | Leucine (LEU) | CD1, CD2 |
|  | Isoleucine (ILE) | CG2, CD1 |
|  | Proline (PRO) | CD |
| Aromatic | Phenylalanine (PHE) | CZ |
|  | Tyrosine (TYR) | CZ, OH |
|  | Tryptophan (TRP) | NE1, CH2 |
| Polar, Uncharged | Serine (SER) | OG |
|  | Threonine (THR) | OG1, CG2 |
|  | Cysteine (CYS) | SG |
|  | Asparagine (ASN) | OD1, ND2 |
|  | Glutamine (GLN) | OE1, NE2 |
| Sulfur-containing | Methionine (MET) | SD, CE |
| Charged (Acidic) | Aspartic Acid (ASP) | OD1, OD2 |
|  | Glutamic Acid (GLU) | OE1, OE2 |
| Charged (Basic) | Lysine (LYS) | NZ |
|  | Arginine (ARG) | CZ, NH2 |
|  | Histidine (HIS) | ND1, NE2 |

**SM2.2. Ligand graph construction** The ligand graph ($G_{l}$) was constructed to provide a detailed chemical and structural representation.

**Node and edge definition**: The graph is a standard atom-level representation where nodes are heavy atoms and edges are their covalent bonds.

**3D conformer generation**: An initial 3D conformer for each ligand was generated from its SMILES string using the ETKDG method within the RDKit toolkit [12]. The coordinates of this conformer were used as the initial 3D coordinates ($X_{l})$for the graph nodes.

**Node features**: Each atom node is described by a 19-dimensional feature vector generated using RDKit. This vector comprises: (i) an 11-dimensional one-hot vector for element type (C, N, O, S, F, P, Cl, Br, I, H, and ‘Other’), and (ii) an 8-dimensional vector for chemical and topological properties, including atomic degree, formal charge, total number of associated hydrogens, hybridization state (one-hot encoded for SP, SP2, SP3), aromaticity, and ring membership.

**Sensitivity analysis**: For the multi-conformer sensitivity analysis (Supplementary Results; SR2), an ensemble of 20 distinct conformers was generated for each ligand. This was achieved by varying the random seed in the ETKDG parameters while keeping all other graph construction protocols identical to the single-conformer pipeline.

# **SM3. Baseline model implementation and training**

For a comprehensive performance comparison, two state-of-the-art models, DeepREAL [13] and AiGPro [14], were selected as baselines. Both models were implemented using their publicly available code and retrained on our dataset. To ensure a fair comparison with our framework, modifications were made where necessary to align their predictive tasks with our three-class classification objective (non-binder, agonist, and antagonist).

**SM3.1. DeepREAL** DeepREAL is a multi-stage framework natively designed to classify ligands into three categories: agonist, antagonist, and non-binder. We implemented the model using its publicly available source code. The original pipeline involves three stages: (1) self-supervised pretraining of protein sequences using a Transformer-based architecture [15]; (2) training a binary interaction classifier by integrating protein embeddings with ligand features from a Graph Isomorphism Network (GIN) [16]; and (3) combining all embeddings in an MLP to classify ligand activity. As the model’s architecture is already aligned with our three-class classification objective, no architectural modifications were necessary. We retrained the entire model on our dataset following the training procedures detailed in the original publication to establish a direct and fair performance benchmark.

**SM3.2. AiGPro** AiGPro is a multi-task deep learning framework originally designed to predict bioactivity values for agonists (${EC}_{50}$) and antagonists (${IC}_{50}$) via regression. We implemented the model using the provided Docker environment. The model operates through a four-stage pipeline that processes protein multiple sequence alignments and ligand SMILES strings, uses dilated convolutions and a cross-attention mechanism to model interactions, and finally predicts bioactivity with an MLP. To establish a fair comparison with our classification model, we adapted its architecture for three-class classification. Specifically, the final regression output layer of AiGPro was replaced with a new classification head consisting of a fully connected layer with three output nodes (corresponding to non-binder, agonist, and antagonist) followed by a Softmax activation function. Consequently, the model’s loss function was changed from a regression loss to Categorical Cross-Entropy loss. All other core architectural components, were preserved to maintain the integrity of the original model’s feature extraction process. The adapted model was then retrained from scratch on our dataset.

# **SM3.3. 3D-GNN baseline** To distinguish the contribution of GPCRact’s architectural design from the contribution of using 3D structural inputs, we implemented an additional 3D-GNN baseline. This model was designed to provide a direct, controlled comparison by using the identical 3D protein, ligand graphs, feature representations, and data splits as the final GPCRact model. The baseline’s architecture removes all of GPCRact’s specialized components, including the hierarchical (decoupled) task structure, the cross-attention interaction module, the hybrid E(n)-Equivariant Graph Neural Network (EGNN) [17] / self-attention [15] propagation layers, and the gated inter-module signal transfer. Instead, it consists of a single-stage, unified EGNN encoder and a single classification head. In this model, the ligand graph is first encoded and pooled into a single global vector. This vector is then broadcast and added element-wise to the initial node features of all corresponding protein atoms. The resulting ligand-injected protein graph is processed by the unified EGNN encoder. Finally, the output node embeddings are averaged to obtain a global protein vector, which is passed to a three-layer MLP to produce logits for the three activity classes (non-binder, antagonist, agonist). All training hyperparameters (e.g., optimizer, learning rate, dropout, early stopping criteria) and evaluation protocols were identical to those used for the GPCRact model, ensuring that any performance difference (Fig. 6B) is solely attributable to architectural design.

# **SM4. GPCRact model optimization and performance validation**

This section details the systematic optimization of the model architecture and provides comprehensive validation benchmarks, including architectural ablation, stratified performance by receptor class, and generalization tests on unseen families.

**SM 4.1. Hyperparameter optimization** The final hyperparameters for the GPCRact model were determined through a systematic Bayesian optimization search. To justify the architectural choices, we conducted a sensitivity analysis on a stratified subset (20%) of the training data, evaluating the impact of graph density (k-NN), hierarchical depth, and attention capacity on validation performance and computational efficiency (Table S4). Based on this analysis, the optimal parameters were selected to maximize the three-class Balanced Accuracy while ensuring computational tractability.

# **Table S4**. Hyperparameter sensitivity and efficiency analysis.

| Category | Value | BAcc (Mean$\pm$Std) | Peak Memory (GB) | Runtime (s/epoch) |
| --- | --- | --- | --- | --- |
| **k-NN Size** | 32 | 0.746$\pm$0.025 | 3.0 | 242 |
|  | **64** | **0.757**$\boldsymbol{\pm}$**0.016** | **5.4** | **279** |
|  | 128 | 0.749$\pm$0.016 | 10.4 | 384 |
|  | 256 | N/A (Diverged) | N/A | N/A |
| **EGNN Depth**  *(Global Propagation)* | 2 | 0.751$\pm$0.016 | 4.6 | 244 |
|  | **4** | **0.762**$\boldsymbol{\pm}$**0.012** | **6.8** | **319** |
|  | 5 | 0.751$\pm$0.017 | 8.5 | 366 |
| **Attention Layers**  *(Global Propagation)* | 2 | 0.744$\pm$0.015 | 8.0 | 311 |
|  | **3** | **0.758**$\boldsymbol{\pm}$**0.009** | **5.2** | **279** |
|  | 5 | 0.746$\pm$0.022 | 5.7 | 299 |
| **Attention Heads** | 2 | 0.751$\pm$0.016 | 6.6 | 315 |
|  | **4** | **0.756**$\boldsymbol{\pm}$**0.013** | **5.2** | **276** |
|  | 8 | 0.764$\pm$0.011 | 7.0 | 315 |

The final hyperparameters used for all experiments reported in this study are detailed in Table S5. The model was trained using the AdamW optimizer and a multi-task loss function with early stopping based on the validation set performance.

# **Table S5**. Final hyperparameters for the GPCRact model.

| Category | Parameter | Value |
| --- | --- | --- |
| **Model Architecture** | Hidden dimension | 128 |
|  | Interaction EGNN layers (Protein & Ligand) | 4 |
|  | Propagation EGNN layers | 4 |
|  | Propagation Transformer layers | 3 |
|  | Attention heads | 4 |
| **Training & Optimization** | Optimizer | AdamW |
|  | Learning rate | $1x{10}^{-4}$ |
|  | Weight decay | $1x{10}^{-5}$ |
|  | Dropout rate | 0.4 |
|  | Batch size (per GPU) | 16 |
|  | Early stopping patience | 10 epochs |
| **Loss Functions** | Activity loss weight $(\lambda_{activity})$ | 1.0 |
|  | Binding false-negative penalty $(w_{FN})$ | 1.5 |
| **Inference Logic** | Primary binding probability threshold | 0.5 |
|  | Uncertainty range for rescue logic | [0.4, 0.5] |
|  | Confidence threshold for rescue logic ($\tau_{conf}$) | 0.95 |

**SM4.2. Decomposed performance evaluation** To validate the effectiveness of the chosen hierarchical architecture, we performed a decomposed performance evaluation. Specifically, we compared the decoupled hierarchical model against the shared-representation multi-task model across two distinct subtasks: binding prediction and conditional activity prediction (Table S6).

# **Table S6**. Decomposed performance of architecture variants on binding and activity tasks.

|  | Task 1: Binding Prediction | | | Task 2: Conditional Activity Prediction (on True Binders) | | |
| --- | --- | --- | --- | --- | --- | --- |
| Model Architecture | BAcc | Non-binder Recall | Binder Recall | BAcc | Antagonist Recall | Agonist Recall |
| Shared-representation (1b) | 0.733 | 0.682 | 0.784 | 0.768 | 0.791 | 0.745 |
| **Hierarchical (1c)** | **0.765** | **0.729** | **0.801** | **0.795** | **0.815** | **0.774** |

*Note: The Unified Single-Head model (1a) is excluded from this decomposed analysis as its architecture inherently conflates binding and activity predictions into a single output vector, precluding task-specific evaluation.*

**SM4.3. Stratified performance by receptor class** To assess the model’s robustness against the inherent class imbalance of the training data (predominantly Class A), we performed a stratified performance evaluation across receptor families. We analyzed the test set performance for Classes A, B1, C, and F, excluding Class T2 due to insufficient sample size. As detailed in Table S7, the model demonstrated consistent generalization across all tested families, with the structure-aware architecture maintaining high accuracy even for underrepresented and structurally complex targets like Class C.

# **Table S7**. Stratified performance evaluation across GPCR classes.

| Target Class | Data Portion | Non-binder Recall | Antagonist Recall | Agonist Recall | BAcc |
| --- | --- | --- | --- | --- | --- |
| **Class A** (Rhodopsin) | 93% | 0.793 | 0.841 | 0.811 | 0.815 |
| **Class B1** (Secretin) | 3.1% | 0.765 | 0.920 | 0.781 | 0.822 |
| **Class C** (Glutamate) | 3.3% | 0.810 | 0.832 | 0.765 | 0.802 |
| **Class F** (Frizzled) | 0.5% | - | 0.865 | 0.745 | 0.805* |

*Note: The analysis for Class F was limited to binders due to the absence of confirmed non-binders.*

**SM4.4. Generalization to unseen receptor targets (Receptor-level and Family-level)** To rigorously assess the model’s structural generalizability beyond the standard scaffold-split, we conducted out-of-distribution (OOD) benchmarks at two levels. First, we performed a Leave-One-Receptor-Out (LORO) validation on representative targets from six major families. In this setting, where other members of the same family were retained in the training set, the model achieved a Balanced Accuracy of 0.63–0.77 (Table S8), demonstrating the ability to transfer learned structural motifs within a family. Second, to test the limit of generalization, we conducted a Leave-One-Family-Out (LOFO) cross-validation. In this rigorous evaluation, all receptors belonging to a specific family were strictly excluded. As summarized in Table S9, although a performance drop was observed due to the significant domain shift in topological manifolds, the model maintained predictive power above the random baseline (0.50) across most families, confirming its ability to capture conserved biophysical activation mechanisms beyond simple pattern matching.

# **Table S8**. Generalization performance on unseen receptors (LORO).

| Metric | CHRM2 | HTR2A | DRD2 | ADORA2A | ADRB2 | CNR1 |
| --- | --- | --- | --- | --- | --- | --- |
| Binding  BAcc | 0.755 | 0.681 | 0.712 | 0.594 | 0.572 | 0.572 |
| Activity BAcc | 0.779 | 0.745 | 0.659 | 0.794 | 0.638 | 0.671 |

# **Table S9**. Generalization performance on unseen receptor families (LOFO).

| Metric | Acetylcholine | Serotonin | Dopamine | Adenosine | Adrenergic | Cannabinoid |
| --- | --- | --- | --- | --- | --- | --- |
| Binding  BAcc | 0.618 | 0.599 | 0.591 | 0.565 | 0.589 | 0.607 |
| Activity BAcc | 0.540 | 0.566 | 0.528 | 0.553 | 0.558 | 0.443 |

# **SM5. Classification and Regression Tree (CART) model for structural signal analysis**

This analysis was designed to test the hypothesis that 3D structural features alone contain a deterministic signal sufficient for predicting a GPCR’s functional state, independent of complex deep learning architectures.

**SM5.1. Dataset and feature engineering** The dataset comprised 617 high-resolution GPCR structures (covering both agonist-bound and antagonist-bound states) curated from GPCRactDB. For each structure, a 443-dimensional feature vector was engineered, consisting of two distinct categories of 3D-derived metrics:

- Conformational state features: These features represent the final geometric state of the receptor. They were calculated as the C$\alpha$-C$\alpha$Euclidean distances between all pairs of residues belonging to the seven TM helices. This resulted in a comprehensive set of intra-receptor distance metrics capturing the relative orientation of the core structural elements.
- Conformational displacement features: These features quantify the conformational change from a reference unbound state. For each receptor, a single highest-resolution apo structure was selected as its reference. In cases where no experimental apo structure was available, the AlphaFold2 [18] model was used as a substitute, an approach validated by our structural analysis detailed in SM7. All other structures for that receptor were superimposed onto this reference structure based on their TM domain C$\alpha$atoms. The displacement of each residue’s C$\alpha$atom from its position in the reference apo state was then calculated and used as a feature.

**SM5.2. Model implementation and training** A CART [19] model was implemented using the DecisionTreeClassifier class from the Scikit-learn [20] library in Python. The objective was to classify each structure’s state as agonist-bound or antagonist-bound. To prioritize interpretability and prevent overfitting, the tree’s maximum depth (max_depth) was limited to 5, and the minimum number of samples required to split a node (min_samples_split) was set to 10. The model was trained on the entire dataset of 617 structures, as the goal was not to test generalization but to assess the inherent information content of the structural features themselves. The Gini impurity was used as the criterion for measuring the quality of a split.

# **SM6. Mechanistic interpretability analysis protocol**

This section details the protocols used to extract, quantify, and visualize the model’s learned attention weights to provide mechanistic insight.

**SM6.1. Self-attention weight extraction** To analyze the model’s learned mechanisms, we extracted the self-attention weights from the trained GPCRact model. Specifically, for each prediction on the test set, we retrieved the attention matrix$A\in\mathbb{R}^{NxN}$(where$N$is the number of atomic nodes in the protein graph) from the last TransformerEncoderLayer of the Allosteric Propagation Module. The weights from the multiple attention heads were averaged to produce a single matrix representing the final global interaction map learned by the model for that specific protein-ligand pair.

**SM6.2. Quantification of residue-level importance** The atomic-level attention matrix was converted to a residue-level matrix by aggregating the attention weights corresponding to the atoms of each residue. From this residue-level matrix, a raw importance score$I_{i}$, was calculated for each residue$i$by summing all the attention it receives from other residues$j$in the protein, defined as:$I_{i}=\sum_{j} A_{ji}$. To enable robust comparison across different proteins, these raw scores were converted to ranks within each protein. The ranks were then normalized to a percentile scale (0.0 to 1.0) to yield the final Self-Attention Importance.

**SM6.3. Group analysis of functional motifs** For quantitative analysis, residues were categorized into functionally significant groups based on the Ballesteros-Weinstein (BW) numbering scheme [21]. We defined several key functional motifs, including the DRY motif (R3.50) and the NPxxY motif (Y7.53), along with a negative control group comprising residues in flexible loops and termini. The mean Self-Attention Importance was then calculated for the residues within each group to assess the model’s learned focus on known functional regions.

**SM6.4. Causal validation via *in silico* point mutation** To validate that the model’s learned attention (Fig. 7) reflects physical causality rather than mere correlation, we performed a *in silico* point mutation study. We hypothesized that if GPCRact effectively learned the allosteric mechanism, its prediction should fail when a critical, function-abolishing mutation is introduced. We selected the R3.50A mutation, a canonical activation-dead mutant known to disrupt G-protein coupling despite allowing agonist binding [22]. We used the wild-type apo structure of the$\beta_{2}$-adrenergic receptor (ADRB2). Using PyMOL [23], we computationally replaced Arginine (R) at position 3.50 with Alanine (A), removing its critical sidechain atoms. This mutant structure was then processed through the identical graph construction pipeline as the wild-type. We fed this new mutant graph into the trained GPCRact model, pairing it with 59 known agonist ligands from our test set. Finally, we compared the resulting predicted binding and agonist probabilities against the baseline predictions from the wild-type structure.

**SM6.5. 3D visualization of allosteric pathways** For qualitative analysis and case studies, a protocol was established to visualize the predicted allosteric communication network by highlighting residues with high attentional importance. For a given prediction, per-residue importance scores were calculated as described above. Residues with an importance score in the top percentile (${95}^{th}$percentile) were designated as key nodes in the predicted allosteric network. The coordinates of these high-importance residues were then mapped onto the receptor’s 3D structure and rendered using PyMOL.

**SM6.6. Comparative analysis protocol: Perturbation Response Scanning (PRS)** To contextualize GPCRact’s learned attention map against a classical biophysical framework, we performed a PRS [24] analysis. We selected PRS as it simulates signal propagation via an Elastic Network Model (ENM) [25] and produces a per-residue importance score. Specifically, we used the PRS sensor profile (sensitivity), which measures a residue’s displacement in response to network-wide perturbations. We analyzed 173 Class A GPCR structures from our dataset, spanning apo, agonist-bound, and antagonist-bound states. For each PDB structure, we used the ProDy [26] library to build a$C\alpha$-based Anisotropic Network Model (ANM) [27] (cutoff=15.0$Å$, gamma=1.0). We then calculated the sensor sensitivity profile for all residues. For the Mean Sensor Profile (Fig. S11), the raw PRS sensitivity scores were converted to percentile ranks within each structure, mapped to functional motifs using the BW numbering scheme, and averaged by receptor family.

**SM6.7. Ligand-protein cross-attention analysis** To identify the ligand molecular features driving functional classification, we analyzed the cross-attention weights from the Stage 1 Interaction Module using ADRB2 as a representative case study due to its chemical diversity in the test set. We extracted the protein-to-ligand attention matrix and averaged it across heads to obtain a global interaction map. The total attention received by each ligand atom was quantified by summing the weights from all binding site atoms. These atomic importance scores were then paired with their corresponding 19-dimensional chemical feature vectors and ground-truth MoA labels. Finally, aggregating across the entire test set, we calculated the differential mean attention for each chemical feature (e.g., Elem_N, IsAromatic) between agonist and antagonist classes to identify discriminative pharmacophores (Table S10).

# **Table S10**. Differential cross-attention analysis of ligand pharmacophores for ADRB2.

| Feature | Agonist Mean Attention | Antagonist Mean Attention | Differential | Agonist Atom Count | Antagonist Atom Count |
| --- | --- | --- | --- | --- | --- |
| **Elem_S** | 16.216 | 8.016 | **+8.200** | 86 | 46 |
| Elem_P | 0.000 | 0.000 | 0.000 | 0 | 0 |
| Hyb_SP2 | 8.957 | 9.041 | -0.085 | 2372 | 2015 |
| Elem_C | 3.938 | 4.379 | -0.441 | 2813 | 2665 |
| IsAromatic | 5.173 | 5.678 | -0.505 | 1795 | 1558 |
| IsInRing | 6.413 | 7.279 | -0.866 | 2288 | 2050 |
| Elem_F | 9.973 | 12.410 | -2.437 | 45 | 14 |
| Hyb_SP3 | 11.037 | 13.805 | -2.768 | 1340 | 1438 |
| Elem_O | 24.944 | 28.042 | -3.099 | 412 | 385 |
| FormalCharge | 15.436 | 19.943 | -4.507 | 10 | 2 |
| **Elem_N** | 36.674 | 44.988 | **-8.315** | 344 | 324 |
| **Elem_Cl** | 13.489 | 23.557 | **-10.068** | 15 | 20 |

#

# **SM7. Validation of AlphaFold2 structures as proxies for apo states**

Many GPCRs in our dataset lack high-resolution experimental apo structures, which are essential for defining the AS and calculating conformational displacement features. To address this, we evaluated the suitability of AlphaFold2 (AF2) [28] models as proxies for the apo state. We conducted a comparative structural analysis on a subset of 63 GPCRs where high-quality experimental structures for all three states (apo, antagonist-bound, agonist-bound) were available. For each receptor, the AF2-predicted structure (v3, sourced from the AlphaFold Protein Structure Database [18]) was superimposed onto the experimental structures via TM domain C$\alpha$alignment. The Root-Mean-Square Deviation (RMSD) was then calculated to quantify structural similarity.

# **SM8. Proof-of-concept virtual screening and lead prioritization simulation**

To validate the translational relevance of GPCRact, we simulated a two-stage drug discovery workflow using the external test sets for ADRB2 (303 compounds) and CHRM2 (478 compounds).

- Stage 1 (Virtual Screening): To simulate a realistic screening scenario, we constructed a decoy-enriched library by undersampling true binders to achieve a 5:1 non-binder to binder ratio. Performance was evaluated using two standard metrics: (i) the Area Under the ROC Curve (AUC) for overall ranking ability, and (ii) the Enrichment Factor 1% (EF1%) [29], which measures the ratio of true binders found in the top 1% of the ranked list relative to the overall binder rate.
- Stage 2 (Lead Prioritization): To validate functional prioritization—a critical step for lead optimization—we utilized the full test set for each target. Following the model’s hierarchical logic, compounds predicted as binders were identified and subsequently re-ranked based on their Stage 2 agonist probability. Performance was assessed using Precision@10 (P@10), quantifying the fraction of true agonists among the top 10 functionally prioritized compounds.

#

# **SM9. Implementation details and computational environment**

All deep learning models described in this study were implemented using the PyTorch framework (v2.5.1) [30]. Training and evaluation were performed on a Linux-based workstation equipped with NVIDIA Tesla V100-SXM2-32GB GPUs. The key software packages and their versions used to ensure the reproducibility of our results are as follows:

- Core Libraries: Python (v3.9.23), CUDA (v12.1), NumPy (v1.23.5), Pandas (v1.5.3).
- Machine Learning: Scikit-learn (v1.3.2).
- Chemoinformatics: RDKit (v2024.3.6).

**Supplementary Results**

# **SR1. Logical consistency analysis of hierarchical predictions**

To validate the logical dependency of the hierarchical architecture, we analyzed activity predictions conditional on binding outcomes (Fig. S8). For correctly identified binders (True Positives), activity predictions were highly accurate (87.0%). Conversely, for false positive binders—where no physical interaction exists—predictions collapsed to a near-random distribution (agonist 60.2% vs. antagonist 39.8%). This indicates that when the binding signal is incorrect, the downstream functional module appropriately lacks the discriminative information required to make a confident prediction, confirming the model’s logical integrity.

# **SR2. Sensitivity analysis of ligand conformational variability**

We evaluated the stability of GPCRact’s predictions across a multi-conformer ensemble (20 distinct conformers per ligand for 2,000 test pairs). The model demonstrated exceptional robustness to input structural uncertainty:

- Binding Prediction Stability: True binders were consistently identified regardless of the input conformer, with 99.9% of variants correctly retained as binders.
- Activity Prediction Consistency: The functional classification (agonist vs. antagonist) showed high stability, achieving an average intra-pair concordance rate of 99.8%.
- Prediction Variance: The model exhibited negligible sensitivity to coordinate perturbations (mean standard deviation of agonist probability = 0.004).
- Ensemble Performance: The ensemble-averaged accuracy (0.825) was statistically equivalent to the single-conformer baseline (0.824).

These results confirm that GPCRact extracts robust, invariant 3D pharmacophoric features rather than overfitting to specific coordinates of a single generated conformer.

# **SR3. Comparative analysis of allosteric maps and MoA-specific attention**

To contextualize the mechanistic insights from GPCRact (Fig. 7), we performed a quantitative benchmark against the classical biophysical PRS method. First, we analyzed the mean PRS sensor profile (Fig. S11), averaged across all available structures (apo, agonist, and antagonist-bound). This map assigns the highest importance to the Flexible Loop/Terminus regions, consistent with their low structural constraints. This confirms that the PRS map primarily reflects the intrinsic physical flexibility of the receptor structure. Second, we analyzed the state-dependent differential map (agonist-bound vs. antagonist-bound) to investigate potential functional discriminability. This analysis showed that PRS profiles do not exhibit consistent, motif-level directional changes across families. This suggests that the map of intrinsic physical dynamics, even when state-dependent, is distinct from the predictive functional map required for classification. GPCRact, designed for this predictive task, captures this ligand-conditioned functional map. Fig. S12 shows that the model assigns differential importance to key motifs (e.g., DRY, NPxxY) specifically depending on whether the ligand is an agonist or antagonist. These results highlight the complementary nature of the two approaches: PRS characterizes intrinsic structural dynamics, whereas GPCRact learns the conditional feature weighting necessary for functional prediction.

# **SR4. Case study: Utility in virtual screening and lead prioritization**

We simulated a two-stage drug discovery workflow to demonstrate translational utility. In the Stage 1 Virtual Screening (5:1 decoy imbalance), the model effectively discriminated binders, achieving high AUCs (0.89 for ADRB2, 0.90 for CHRM2) and near-optimal EF1% scores (5.75 and 5.80). In the Stage 2 Lead Prioritization, the model successfully ranked compounds by functional efficacy. For ADRB2, it achieved a perfect Precision@10 of 1.00 (identifying 10 true agonists in the top 10). For CHRM2, it maintained strong prioritization with a Precision@10 of 0.70. These results validate the hierarchical framework’s ability to filter non-binders and effectively prioritize functional leads.


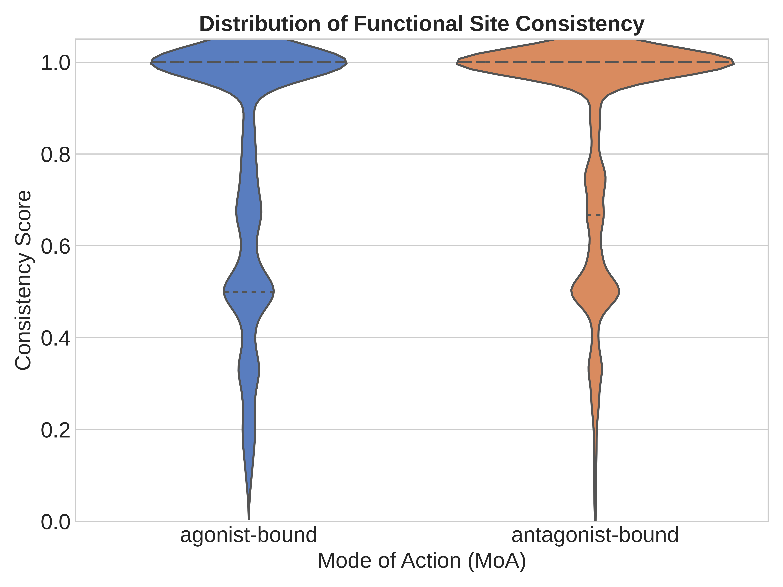
**Supplementary Figures**

# **Figure S1**. **Validation of functional site consistency.** Violin plots showing the distribution of consistency scores for residues constituting the functional sites (consensus Binding Site and consensus Allosteric Site). The analysis spans all available holo structures for each receptor, grouped by the ligand’s MoA. The consistency score represents the fraction of holo structures in which a residue is identified as part of the functional site. Median scores approaching 1.0 confirm that these sites are highly stable and conserved features across diverse conformational states, validating the use of a unified, consensus-based site definition.

# **
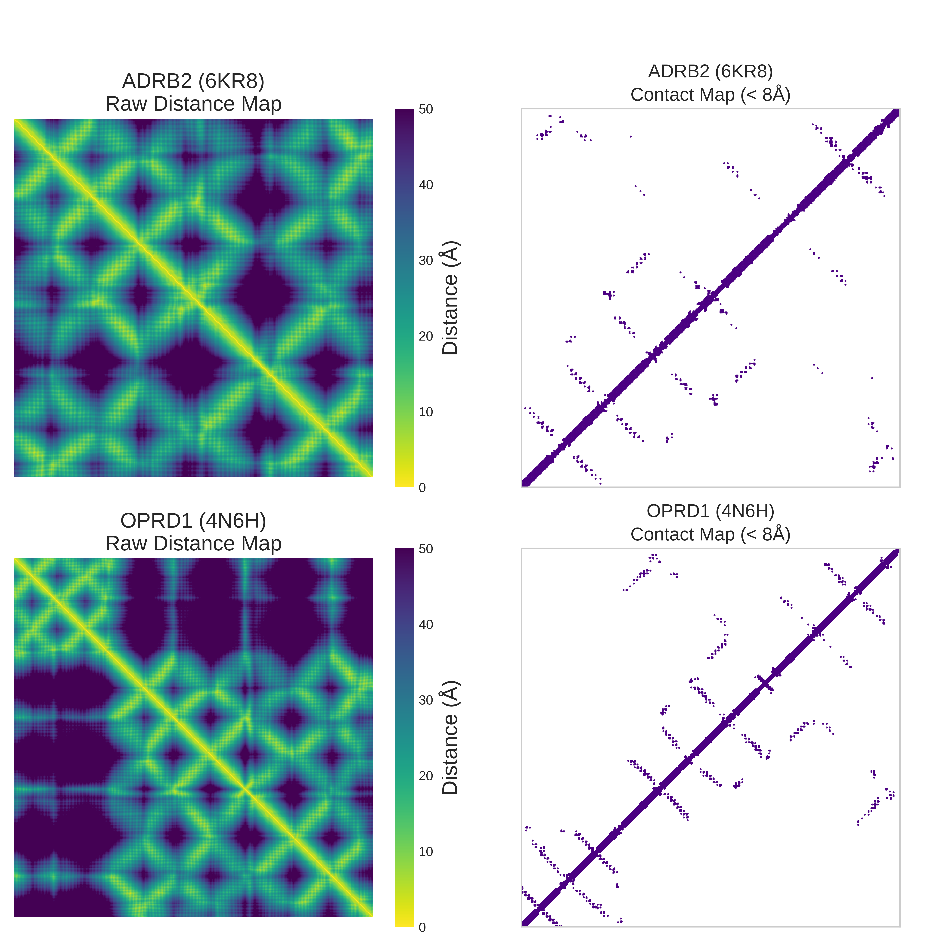
****Figure S2. Comparison of 3D raw distance maps and 2D contact maps** Illustration of the spatial information loss when reducing a 3D structure to a 2D representation. For two representative GPCRs (ADRB2, PDB: 6KR8[23] and OPRD1, PDB: 4N6H[24]), the Raw Distance Maps (left) display the complete pairwise C$\alpha$-C$\alpha$distances, representing the rich geometric information inherent in the 3D structure. In contrast, the contact maps (right) are sparse, binary representations showing only residue pairs within an 8.0$Å$threshold. This visual comparison demonstrates the substantial loss of medium- and long-range spatial information that occurs in simplified representations. This comparison highlights the substantial loss of spatial information in simplified representations, underscoring the rationale for GPCRact’s direct use of 3D atomic coordinates.

# **Figure S3. Validation of AlphaFold2 (AF2) structures as apo state proxies.** Box plots show the C$\alpha$-RMSD between AF2-predicted structures and experimentally determined structures in apo, antagonist-bound, and agonist-bound states for 63 GPCRs. The analysis confirms that AF2 structures are significantly closer to the experimental apo conformation than to active-like states. The dashed red line indicates a 2.0$Å$RMSD threshold, a common metric for high structural similarity. P-values from unpaired two-sample t-tests, relative to the AF2–apo distribution, are provided.


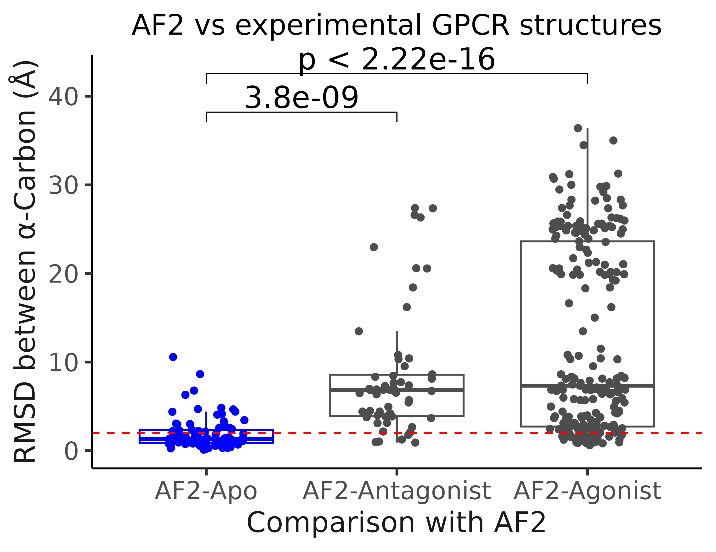


RMSD 2.0$Å$ (threshold)

# **
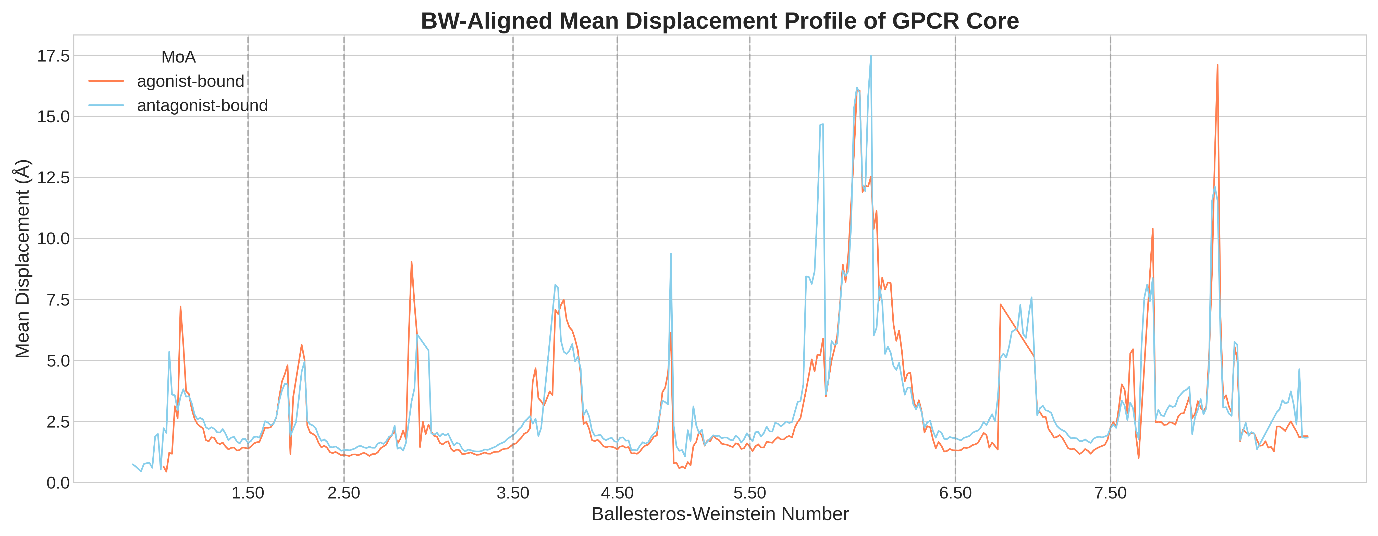
****Figure S4. BW-aligned mean C**$\boldsymbol{\alpha}$**displacement profiles for agonist- vs. antagonist-bound GPCRs.** Comparison of conserved conformational changes induced by agonists versus antagonists across the GPCR transmembrane core. The plot shows the mean C$\alpha$displacement for each residue, averaged across all agonist-bound (orange) and antagonist-bound (blue) structures relative to their respective reference apo states, aligned by the Ballesteros-Weinstein (BW) numbering. The profiles reveal that agonists consistently elicit significantly larger conformational changes in regions critical for G-protein coupling, particularly at the intracellular ends of TM5 and TM6 (BW 5.50–6.50). This demonstrates that a conserved, MoA-dependent dynamic signature is encoded in the 3D structure.

# **
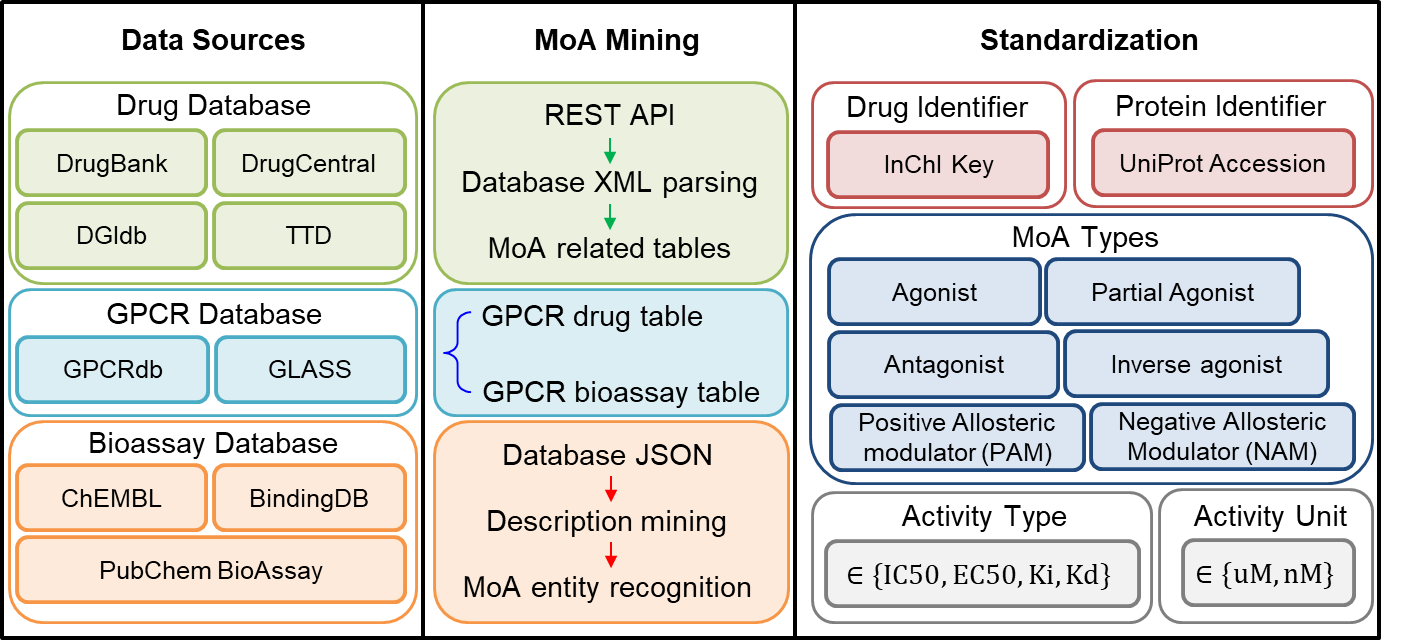
Figure S5. Overall workflow for the construction of the GPCRactDB.** The pipeline integrates data from three primary categories of public repositories: approved drug databases, GPCR-specific databases, and large-scale bioassay databases. Data from these diverse sources are processed through parallel workflows—including REST API parsing for structured data and a dictionary-based text mining pipeline for unstructured bioassays—to extract and harmonize MoA annotations. All curated data are subsequently unified through a final standardization process. A complete, step-by-step description of this pipeline, including all data sources and curation rules, is provided in Supplementary Methods (SM1).

# **
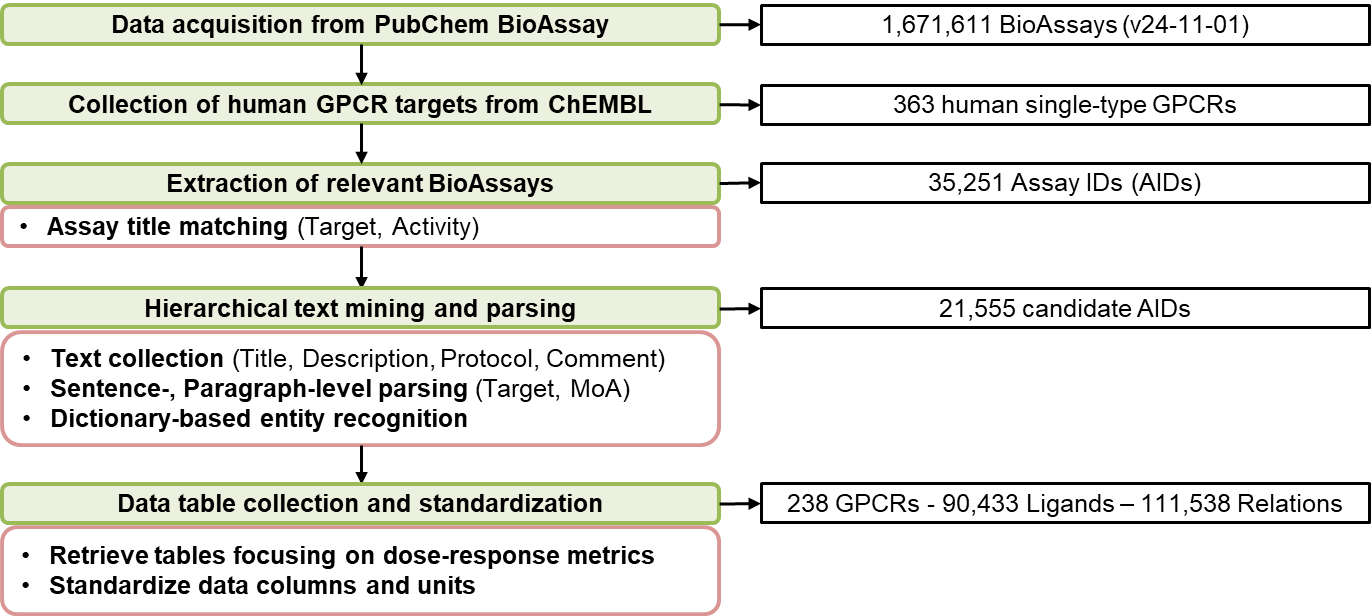
Figure S6. Hierarchical text mining pipeline for PubChem BioAssay curation.** Illustration of the multi-stage process used to extract, filter, and standardize functional activity data from the unstructured PubChem BioAssay database. The workflow begins by identifying all human GPCR-related assays, which are progressively filtered from an initial set of over 1.6 million bioassays down to 35,251 relevant Assay IDs (AIDs) through target-based matching. These candidates subsequently undergo a hierarchical text mining process, employing dictionary-based Named Entity Recognition (NER) on assay descriptions and protocols, to yield 21,555 AIDs with sufficient information for MoA annotation. The final stage involves the collection and standardization of dose-response data, ultimately contributing 111,538 high-confidence ligand-GPCR interactions to the final GPCRactDB.

# **
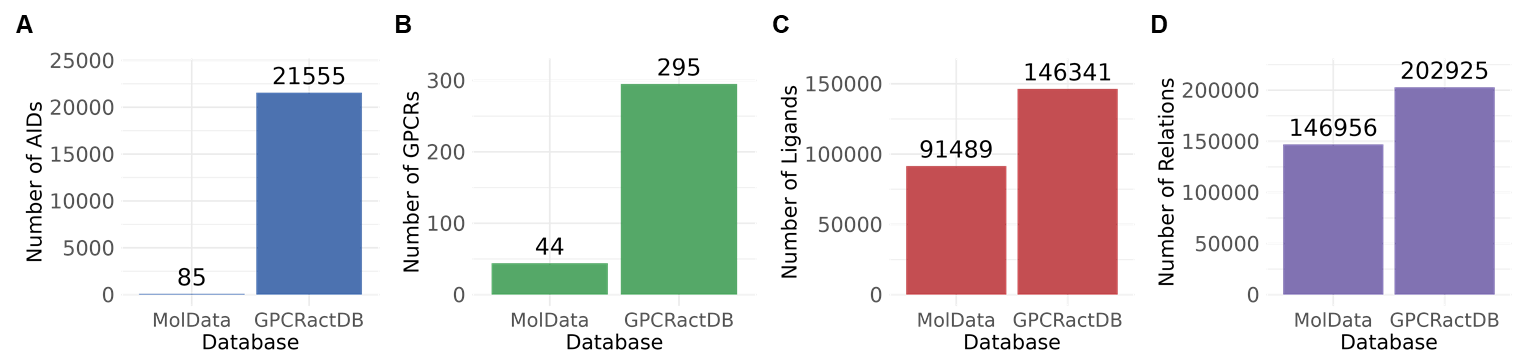
Figure S7. Quantitative scale comparison of GPCRactDB and the MolData database.** Bar charts comparing the scale and scope of GPCRactDB against MolData [31], a prior database for GPCR activity. The four bar charts compare the databases across key metrics (from left to right): (A) number of curated AIDs, (B) number of unique GPCRs, (C) number of unique ligands, and (D) total number of ligand-GPCR relations. The results demonstrate the significant advancement of GPCRactDB in both scale and functional specificity. While the prior database was limited by a smaller set of assays with mixed data types and lacked unified activity criteria, GPCRactDB was constructed using a systematic pipeline focused exclusively on ligand-induced functional activity, resulting in a more comprehensive resource for predictive modeling.

# **
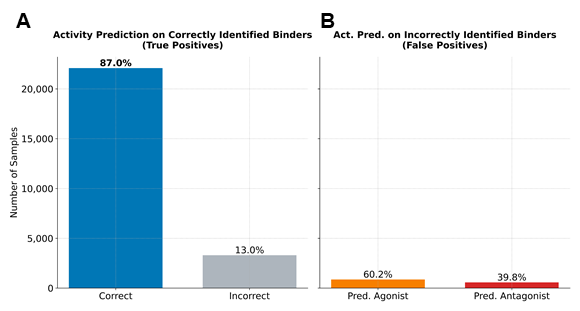
Figure S8. Logical consistency of activity predictions conditional on binding outcomes.** Bar charts displaying the distribution of predicted activity classes for (A) correctly identified binders (True Positives) and (B) incorrectly identified binders (False Positives). The stark contrast between the high-confidence classification in TP samples and the random distribution in FP samples confirms that the model’s functional predictions are logically dependent on valid ligand-receptor interactions.

# **
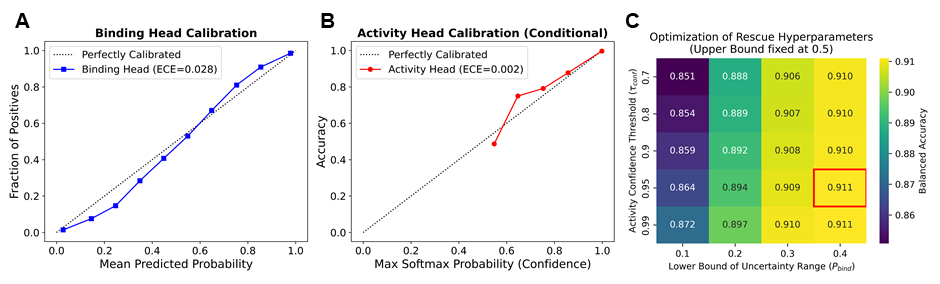
Figure S9. Statistical validation and hyperparameter optimization of the confidence-based rescue logic.** Calibration plots for the Binding Head (A) and Activity Head (B). While the Binding Head shows standard calibration (Expected Calibration Error [32], ECE=0.028), the Activity Head demonstrates exceptional reliability (ECE=0.002) on conditional binders. This empirical reliability justifies using high-confidence activity scores to correct uncertain binding predictions. (C) Heatmap visualization of the grid search optimization on the validation set. The analysis identifies the optimal configuration of uncertainty ranges and confidence thresholds that maximizes Balanced Accuracy.

# **
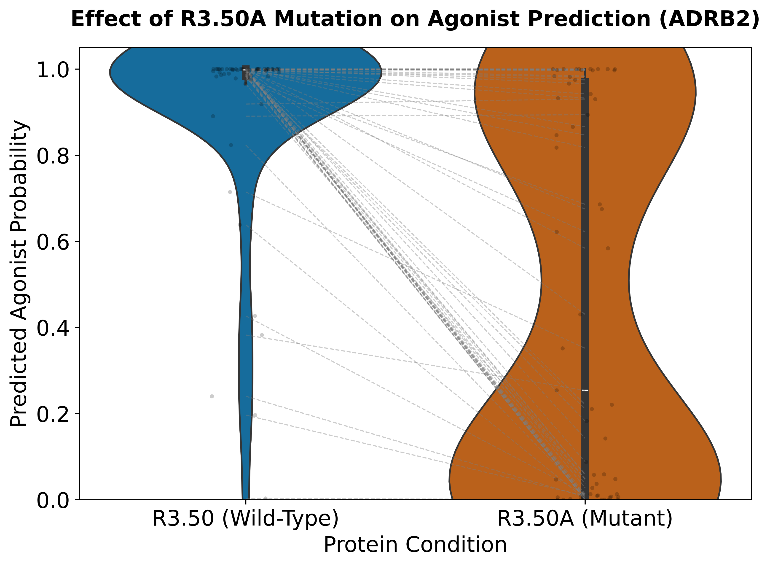
Figure S10. Causal validation of GPCRact via *in silico* point mutation of the R3.50 activation switch.** Violin plots comparing the distribution of predicted agonist probabilities for 59 known test-set agonists when paired with the Wild-Type ADRB2 receptor (blue) versus the *in silico* R3.50A Mutant structure (orange). The R3.50A mutation, which is known to abolish G-protein coupling, caused the model’s mean agonist prediction to collapse from 0.916 (WT) to 0.457 (Mutant). This drastic reduction provides strong evidence that GPCRact has learned the physical causality of activation, rather than relying on spurious correlations.

# **
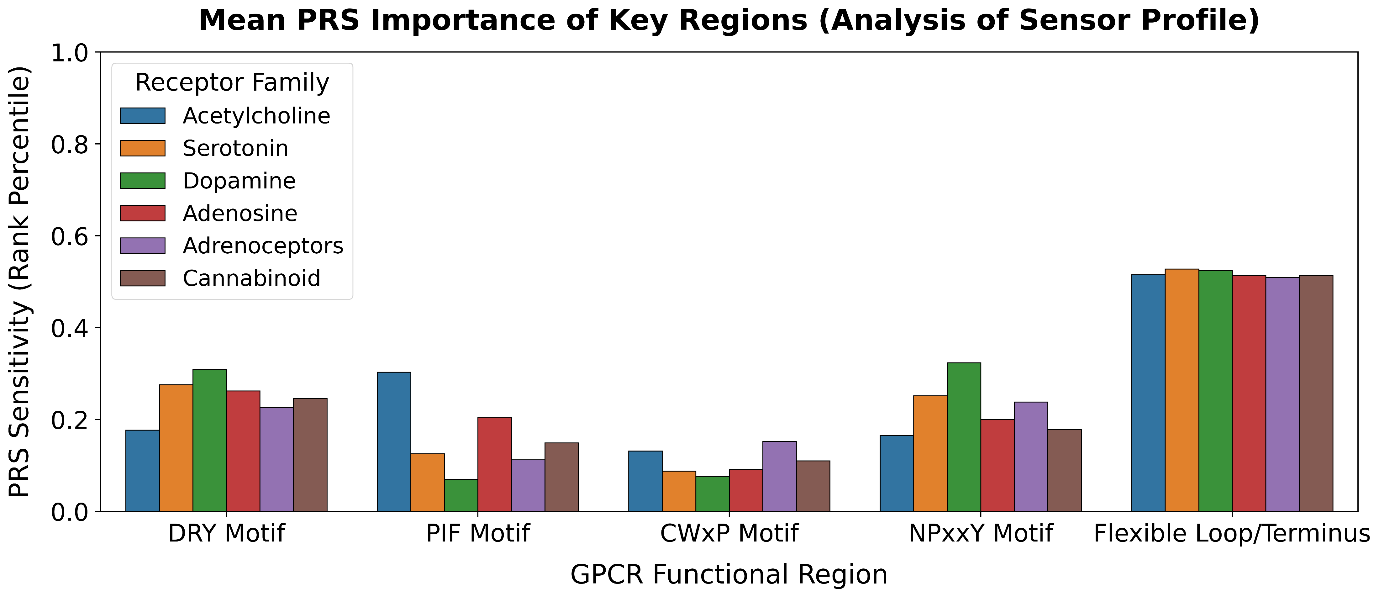
Figure S11. Mean PRS sensor profile of key functional regions.** Bar chart displaying the mean Perturbation Response Scanning (PRS) sensor sensitivity for canonical functional regions. Importance scores were normalized to a percentile rank (0.0 to 1.0) within each PDB structure and averaged by family. The map, reflecting intrinsic physical flexibility, assigns the highest importance to the Flexible Loop/Terminus regions.

# **
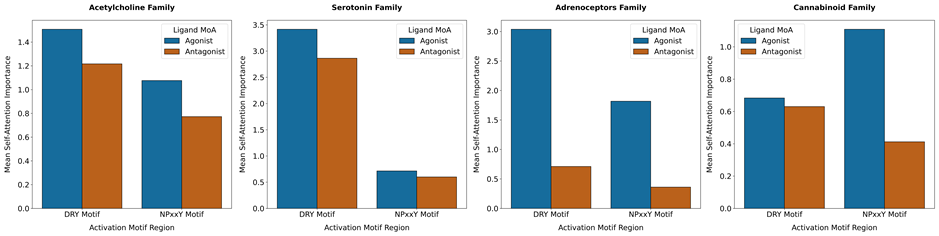
Figure S12. Ligand-dependent (MoA-specific) modulation of attention patterns.** Comparison of mean self-attention importance for key activation motifs (DRY, NPxxY) for agonist and antagonist ligands across four receptor families. GPCRact dynamically shifts importance to core motifs based on the ligand’s specific functional outcome, assigning significantly higher attention to activation switches (DRY, NPxxY) in the presence of agonists.

**References**

1. Knox C, Wilson M, Klinger CM et al. DrugBank 6.0: the DrugBank knowledgebase for 2024, Nucleic acids research 2024;52:D1265-D1275.

2. Ursu O, Holmes J, Knockel J et al. DrugCentral: online drug compendium, Nucleic acids research 2016:gkw993.

3. Cannon M, Stevenson J, Stahl K et al. DGIdb 5.0: rebuilding the drug–gene interaction database for precision medicine and drug discovery platforms, Nucleic acids research 2024;52:D1227-D1235.

4. Chen X, Ji ZL, Chen YZ. TTD: therapeutic target database, Nucleic acids research 2002;30:412-415.

5. Herrera LPT, Andreassen SN, Caroli J et al. GPCRdb in 2025: adding odorant receptors, data mapper, structure similarity search and models of physiological ligand complexes, Nucleic acids research 2025;53:D425-D435.

6. Chan WK, Zhang H, Yang J et al. GLASS: a comprehensive database for experimentally validated GPCR-ligand associations, Bioinformatics 2015;31:3035-3042.

7. Wang Y, Bryant SH, Cheng T et al. Pubchem bioassay: 2017 update, Nucleic acids research 2017;45:D955-D963.

8. Gaulton A, Hersey A, Nowotka M et al. The ChEMBL database in 2017, Nucleic acids research 2017;45:D945-D954.

9. Gilson MK, Liu T, Baitaluk M et al. BindingDB in 2015: a public database for medicinal chemistry, computational chemistry and systems pharmacology, Nucleic acids research 2016;44:D1045-D1053.

10. Harding SD, Sharman JL, Faccenda E et al. The IUPHAR/BPS Guide to PHARMACOLOGY in 2018: updates and expansion to encompass the new guide to IMMUNOPHARMACOLOGY, Nucleic acids research 2018;46:D1091-D1106.

11. Consortium U. UniProt: a hub for protein information, Nucleic acids research 2015;43:D204-D212.

12. Landrum G. RDKit: A software suite for cheminformatics, computational chemistry, and predictive modeling, Greg Landrum 2013;8:5281.

13. Cai T, Abbu KA, Liu Y et al. DeepREAL: a deep learning powered multi-scale modeling framework for predicting out-of-distribution ligand-induced GPCR activity, Bioinformatics 2022;38:2561-2570.

14. Brahma R, Moon S, Shin J-M et al. AiGPro: a multi-tasks model for profiling of GPCRs for agonist and antagonist, Journal of Cheminformatics 2025;17:12.

15. Vaswani A, Shazeer N, Parmar N et al. Attention is all you need, Advances in neural information processing systems 2017;30.

16. Xu K, Hu W, Leskovec J et al. How powerful are graph neural networks?, arXiv preprint arXiv:1810.00826 2018.

17. Satorras VG, Hoogeboom E, Welling M. E (n) equivariant graph neural networks. In: International conference on machine learning. 2021, p. 9323-9332. PMLR.

18. Varadi M, Bertoni D, Magana P et al. AlphaFold Protein Structure Database in 2024: providing structure coverage for over 214 million protein sequences, Nucleic acids research 2024;52:D368-D375.

19. Breiman L, Friedman J, Olshen RA et al. Classification and regression trees. Chapman and Hall/CRC, 2017.

20. Pedregosa F, Varoquaux G, Gramfort A et al. Scikit-learn: Machine learning in Python, the Journal of machine Learning research 2011;12:2825-2830.

21. Ballesteros JA, Weinstein H. [19] Integrated methods for the construction of three-dimensional models and computational probing of structure-function relations in G protein-coupled receptors. Methods in neurosciences. Elsevier, 1995, 366-428.

22. Feng W, Song Z. Effects of D3. 49A, R3. 50A, and A6. 34E mutations on ligand binding and activation of the cannabinoid-2 (CB2) receptor, Biochemical pharmacology 2003;65:1077-1085.

23. Schrodinger L. The PyMOL molecular graphics system, Version 2015;1:8.

24. Atilgan C, Atilgan AR. Perturbation-response scanning reveals ligand entry-exit mechanisms of ferric binding protein, PLoS computational biology 2009;5:e1000544.

25. Gerek ZN, Ozkan SB. Change in allosteric network affects binding affinities of PDZ domains: analysis through perturbation response scanning, PLoS computational biology 2011;7:e1002154.

26. Bakan A, Meireles LM, Bahar I. ProDy: protein dynamics inferred from theory and experiments, Bioinformatics 2011;27:1575-1577.

27. Atilgan A, Durell S, Jernigan R et al. Elastic network model for protein structural dynamics, Biophys J 2001;80:505-515.

28. Jumper J, Evans R, Pritzel A et al. Highly accurate protein structure prediction with AlphaFold, Nature 2021;596:583-589.

29. Truchon J-F, Bayly CI. Evaluating virtual screening methods: good and bad metrics for the “early recognition” problem, Journal of chemical information and modeling 2007;47:488-508.

30. Paszke A, Gross S, Chintala S et al. Automatic differentiation in pytorch 2017.

31. Keshavarzi Arshadi A, Salem M, Firouzbakht A et al. MolData, a molecular benchmark for disease and target based machine learning, Journal of Cheminformatics 2022;14:10.

32. Guo C, Pleiss G, Sun Y et al. On calibration of modern neural networks. In: International conference on machine learning. 2017, p. 1321-1330. PMLR.
